# Addressing Behavioral Barriers to COVID-19 Testing With Health Literacy–Sensitive eHealth Interventions: Results From 2 National Surveys and 2 Randomized Experiments

**DOI:** 10.2196/40441

**Published:** 2023-06-29

**Authors:** Carissa Bonner, Carys Batcup, Erin Cvejic, Julie Ayre, Kristen Pickles, Tessa Copp, Samuel Cornell, Brooke Nickel, Mustafa Dhahir, Kirsten McCaffery

**Affiliations:** 1 School of Public Health Faculty of Medicine and Health University of Sydney Sydney Australia

**Keywords:** behavior change, health literacy, COVID-19, testing, infectious disease, public health

## Abstract

**Background:**

Polymerase chain reaction (PCR) testing for COVID-19 was crucial in Australia’s prevention strategy in the first 2 years of the pandemic, including required testing for symptoms, contact with cases, travel, and certain professions. However, several months into the pandemic, half of Australians were still not getting tested for respiratory symptoms, and little was known about the drivers of and barriers to COVID-19 PCR testing as a novel behavior at that time.

**Objective:**

We aimed to identify and address COVID-19 testing barriers, and test the effectiveness of multiple eHealth interventions on knowledge for people with varying health literacy levels.

**Methods:**

The intervention was developed in 4 phases. Phase 1 was a national survey conducted in June 2020 (n=1369), in which testing barriers were coded using the capability-opportunity-motivation-behavior framework. Phase 2 was a national survey conducted in November 2020 (n=2034) to estimate the prevalence of testing barriers and health literacy disparities. Phase 3 was a randomized experiment testing health literacy–sensitive written information for a wide range of barriers between February and March 2021 (n=1314), in which participants chose their top 3 barriers to testing to view a tailored intervention. Phase 4 was a randomized experiment testing 2 audio-visual interventions addressing common testing barriers for people with lower health literacy in November 2021, targeting young adults as a key group endorsing misinformation (n=1527).

**Results:**

In phase 1, barriers were identified in all 3 categories: capability (eg, understanding which symptoms to test for), opportunity (eg, not being able to access a PCR test), and motivation (eg, not believing the symptoms are those of COVID-19). Phase 2 identified knowledge gaps for people with lower versus higher health literacy. Phase 3 found no differences between the intervention (health literacy–sensitive text for top 3 barriers) and control groups. Phase 4 showed that a fact-based animation or a TikTok-style video presenting the same facts in a humorous style increased knowledge about COVID-19 testing compared with government information. However, no differences were found for COVID-19 testing intentions.

**Conclusions:**

This study identified a wide range of barriers to a novel testing behavior, PCR testing for COVID-19. These barriers were prevalent even in a health system where COVID-19 testing was free and widely available. We showed that key capability barriers, such as knowledge gaps, can be improved with simple videos targeting people with lower health literacy. Additional behavior change strategies are required to address motivational issues to support testing uptake. Future research will explore health literacy strategies in the current context of self-administered rapid antigen tests. The findings may inform planning for future COVID-19 variant outbreaks and new public health emergencies where novel testing behaviors are required.

**Trial Registration:**

Australian New Zealand Clinical Trials Registry ACTRN12621000876897, https://www.anzctr.org.au/Trial/Registration/TrialReview.aspx?id=382318 ; Australian New Zealand Clinical Trials Registry ACTRN12620001355965, https://www.anzctr.org.au/Trial/Registration/TrialReview.aspx?id=380916&isReview=true

## Introduction

### The Role of Polymerase Chain Reaction Testing in COVID-19

The behavior of individuals has been crucial to the control of COVID-19, from self-isolating and testing to vaccination uptake [1]. A key preventive behavior in the early stages of the pandemic was polymerase chain reaction (PCR) testing for COVID-19 [2]. From 2020 to 2021, COVID-19 prevention strategies were often reliant on people getting a PCR test. This could be required when community members had been in contact with a positive case, had COVID-19 symptoms (eg, fever, cough, or sore throat), needed to travel from an outbreak area to another region, or worked in certain professions (eg, health workers). In Australia, community members were required to self-isolate at home until they returned a negative PCR test result, and this test-trace-isolate strategy was used to determine the need for short-term localized restrictions until linked clusters of cases were brought under control [3].

### Testing Barriers

In early 2020, there was little research on COVID-19 testing behaviors, given the very new nature of this issue, so little was known about the barriers to testing or how to address this. Media reports at this time suggested different barriers existed across countries, which was confirmed in subsequent research. For example, countries such as Tanzania had major issues with opportunity barriers in terms of limited access to COVID-19 tests and fake testing kits [4]. Cost was a barrier in other countries, such as the United States, where the government and health insurers did not cover the testing [5], disproportionately affecting certain groups such as immigrant and noncitizen communities, who may also fear financial and legal repercussions from testing positive [6]. Testing was sometimes limited to certain criteria (eg, only if you have symptoms or regardless of exposure to COVID-19 cases) because of the lack of supply or staff resource issues [7]. There were also issues with delivering tests and transporting samples to remote areas [8]. Inadequate communication and low community knowledge about which symptoms require testing and the process to follow also impacted uptake [7].

### The Australian Context

Australia was fortunate to have efficient and free testing widely available from the start of the pandemic, although this varied by location. PCR testing clinics were established nationally, including drive-through options to minimize contact with others and results sent by SMS text messages within a short period [9,10]. However, despite the high accessibility of PCR testing, flu tracking data suggested that many more people had respiratory symptoms than were getting tested [11]. At the time of the study, it was unclear why the uptake was so low, but we hypothesized that COVID-19 testing communication did not address the needs of varying health literacy levels in the community. Similar to many other countries [12], Australian national surveys showed that people with lower health literacy were less likely to know about COVID-19 symptoms and prevention measures [13] and were more likely to agree with misinformation about COVID-19 [14].

### Theoretical Framework

According to the capability-opportunity-motivation-behavior (COM-B) model [15], health prevention behaviors can be conceptualized in terms of 3 main drivers: physical and psychological *capability* (eg, having the physical ability to drive to or walk up the stairs to access a testing center and knowing what to do if you have symptoms), physical and social *opportunity* (eg, the availability of testing centers in your area and social norms that make testing and self-isolation acceptable), and automatic and reflective *motivation* (eg, fear of a painful test and an explicit belief that it is important to get tested for symptoms) [2]. In early 2020, we used this framework as the basis for a new research program on the novel behavior of COVID-19 PCR testing.

### Objective

This program aimed to develop and test eHealth interventions to overcome COVID-19 PCR testing barriers and address the varying health literacy needs of the community. The interventions were developed and evaluated in 4 phases from June 2020 to November 2021.

- Phase 1, in June 2020, aimed to identify the *range* of barriers to COVID-19 testing.
- Phase 2, in November 2020, aimed to estimate the *prevalence* of barriers to COVID-19 testing and to target interventions for the most important issues.
- Phase 3, from February to March 2021, aimed to test the efficacy of providing health literacy–sensitive written information (ie, adapted for people with lower health literacy) for all capability and motivation barriers identified in phase 2, where individuals could view information to make a plan for their top 3 barriers to testing.
- Phase 4, in November 2021, aimed to address design issues in phase 3 and test the efficacy of providing health literacy–sensitive audio-visual interventions (simple animation or TikTok-style video) for a smaller selection of common barriers for people with lower health literacy, identified in phase 2.

## Methods

### Ethics Approval

Ethics approval was obtained from the University of Sydney Human Research Ethics Committee (project number 2020/781), and the experiments were preregistered on the Australia New Zealand Trial Registry (ACTRN12621000876897 [16]; ACTRN12620001355965 [17]). All data were collected and stored anonymously, but participants could provide contact details to receive compensation via points for panel members and prize draws for gift vouchers if recruited via social media.

### Study Design

**Figure 1:**
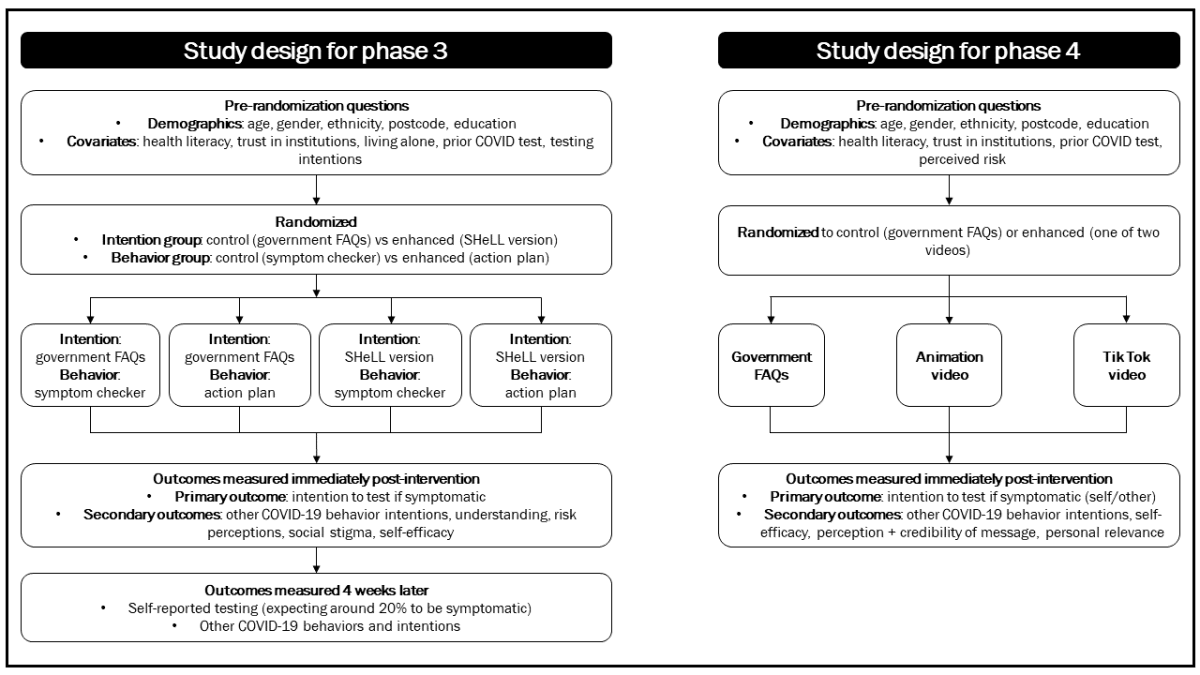
Study design for phases 3 and 4. FAQ: frequently asked question; SHeLL: Sydney Health Literacy Lab.

The eHealth interventions were developed and tested in 4 phases. Phase 1 was a national survey conducted in June 2020, in which testing barriers were elicited and coded using the COM-B framework. Phase 2 was a national survey conducted in November 2020 to estimate the prevalence of testing barriers and health literacy disparities. Phase 3 was a randomized experiment testing health literacy–sensitive written information for a wide range of capability and motivation barriers from February to March 2021, in which participants chose their top 3 barriers to testing to view a tailored intervention. Phase 4 was a randomized experiment testing 2 audio-visual interventions addressing common testing barriers for people with lower health literacy in November 2021, targeting young adults as a key group endorsing misinformation (Figure 1).

### Study Population

Phase 1 was open to any Australian adult who responded to the advertisements on social media. Phases 2 and 3 recruited a nationally representative sample of Australian adults based on age (equal groups above and below the age of 40 years), gender (equal groups for male and female; no quota for other categories), and education (equal groups for university degree and no degree). Phase 4 targeted younger Australian adults (aged <40 years) using the same market research panel.

### Recruitment

Phase 1 used advertisements to target social media users, in which participants entered a prize draw to win a gift voucher. Phases 2 and 3 recruited a nationally representative sample via a market research panel company, where participants received points across multiple studies that they can redeem for various incentives including gift vouchers. Phase 4 involved both social media advertisements and panel recruitment. Different states in Australia were targeted at different times of recruitment so that participants were only recruited when there were very few or no cases in their state (when a test-trace-isolate strategy can be effective for containing spread).

### Sample Size

For each phase, we recruited the following number of people: 1369 in phase 1; 20,349 in phase 2; 1314 in phase 3; and 1527 in phase 4.

### Data Collection

All 4 phases involved piloting with a convenience sample from the target participant group before recruitment to identify and correct any grammar or navigation issues. The interventions were tested by a consumer representative who provided feedback to refine the study materials before formal data collection.

In phase 1, social media users were asked to participate in a series of 10-minute surveys, with COVID-19 testing questions included in June 2020. The New South Wales State Health Department provided a short list of testing barriers to be included along with an open response option for other perceived or experienced barriers. Phase 2 recruited a nationally representative sample, in which eligible panel members were invited to participate through the company’s usual channels. After providing informed consent, participants completed a 10-minute survey, which included selecting COVID-19 testing barriers from a list and ranking them in order of importance.

A similar procedure was used for phases 3 and 4, with recruitment through a market research panel company, but participants were randomized to view different versions of COVID-19 testing information. In phase 3, they were randomized to view written government information or the intervention and completed the outcome questions. Those randomized to the intervention selected 3 relevant barriers, viewed health literacy–sensitive information about those issues, and created a plan for 1 chosen barrier. They were asked to create an action plan to help them overcome their barriers and received weekly reminders with a screenshot of their action plan by email. After 4 weeks, all participants received a 5-minute survey. In phase 4, social media users aged 18-39 years on Facebook and Instagram were targeted with advertisements, and further participants were recruited via the panel company. The following advertisement text was used for social media: “We want to hear from you! Complete a short survey about COVID-19 and be in with the chance to win a $20 gift card.” All participants answered a 10-minute survey. Those randomized to the intervention groups viewed a short audio-visual intervention, whereas those in the control group viewed standard written government information. Access to the outcome questions to complete the survey was enabled after 73 seconds for the animation and 65 seconds for the TikTok (the lengths of the audio-visual intervention) to increase the chance that participants viewed the intervention.

### Outcomes

Survey questions for phases 1 and 2 are provided in Multimedia Appendix 1. In each phase, we measured variables shown to be associated with differences in the understanding of COVID-19 symptoms and prevention measures in our previous research [13,18]: age, gender, language, health literacy, trust, living alone, and prior COVID test. For the trials, our primary outcome was the intention to undergo testing for COVID-19 if symptomatic (measured in a broad way for phase 3 and a more specific way for phase 4 to increase sensitivity). The secondary outcomes included intentions about other prevention behaviors (self-isolation if symptomatic, social distancing of 1.5 m, washing hands regularly, and wearing masks in crowded indoor areas), understanding of messaging, risk perceptions, social stigma, and self-efficacy (ie, confidence in overcoming perceived barriers to testing). In phase 3 only, self-reported prevention behavior and intentions were assessed after 1 month, with our prior survey data suggesting that >20% of participants would experience symptoms over that time (Table 1).

**Table 1:** Primary outcome measures for phases 3 and 4.

Outcome and items		Response options	Phase 3	Phase 4
**Intention to get tested for COVID-19**				
	Over the next 4 weeks I plan to get tested if I have COVID-19 symptoms (cough, sore throat, fever)	1=strongly disagree to 7=strongly agree	✓	✓
	Imagine you woke up with a sore throat tomorrow. Would you get tested straight away?	1=extremely unlikely to 5=extremely likely		✓
	Most people my age would get tested after seeing this information, if they develop symptoms	1=strongly disagree to 7=strongly agree		✓
**Intention to engage in other preventive behaviors**				
	Over the next 4 weeks I plan to...Stay home if I have COVID-19 symptoms (cough, sore throat, fever)/Stay 1.5m away from others that I don’t live with where I can/Wash my hands or use sanitiser to protect me and others from COVID-19/Wear a mask in crowded indoor areas	1=strongly disagree to 7=strongly agree	✓	✓
**Knowledge**				
	Can you name 3 signs or symptoms that are associated with COVID-19?	Written answer	✓	
	When someone has signs that they might have COVID-19 (e.g. a cough or a sore throat), they should...	Wait until their symptoms are bad enough, then get tested/get tested straight away and self-isolate at home until they get their test results/get tested straight away and carry on as normal until they get their test results/self-isolate at home until their symptoms go away	✓	
	If someone has a sore throat and wants to get tested for COVID-19, they should go... (choose all that apply)	To the emergency department/to their GP^a^/to a COVID-19 testing center	✓	
	If someone gets tested for COVID-19, and they need groceries while waiting for their result, they should ... (choose all that apply)	Ask someone to get the groceries for them/wear a mask at the shops/go to the shops quickly to get essentials/order online	✓	
	What are the 6 main COVID-19 symptoms you should get tested for? Select from the list	Sore throat/loss of taste or smell/digestive issues/cough/muscle aches/vomiting/fever/conjunctivitis/runny nose/shortness of breath or difficulty breathing/diarrhea		✓
	When do you need to get tested?	Any mild/slight symptoms/moderate/uncomfortable symptoms/symptoms are severe/disrupt your plans		✓
	When do you need to get tested?	Any length of time with symptoms/symptoms lasting 2 days/symptoms lasting 3 days		✓
	When do you need to get tested?	One or more symptoms in a single day/2 or more symptoms in a single day/3 or more symptoms in a single day		✓
	If you have symptoms of COVID-19, you should get tested when... Select all that apply	There are hotel quarantine cases in your state/there are local cases in your community/there are no local cases in your community		✓
	Should you get tested if you have unusual or new cold-like symptoms that you think are due to: Cold weather/a cold/flu/hayfever/allergies	1=yes definitely to 5=no definitely not		✓

^a^GP: general practitioner.

### Analyses

For phases 1 and 2, participant characteristics and survey question responses were reported descriptively using a content analysis approach for open responses and chi-square tests to compare responses across health literacy levels. In total, 2 researchers mapped the text from open responses to the components of the COM-B model, with discussion to resolve discrepancies. For phases 3 and 4, analyses were conducted using planned contrasts between the intervention arms and control arm, implemented in the regression models. Continuous outcomes were analyzed using linear regression to estimate marginal mean differences, dichotomous outcomes were analyzed using generalized linear models with a modified Poisson approach (log link and robust SEs) to estimate relative risks, and count variables were analyzed using Poisson regression to estimate relative risks. In phase 3, analyses were controlled for age, gender, language, health literacy, trust, living alone, and previous COVID-19 testing. In phase 4, positive baseline intention, age, gender, language, health literacy, trust, and perceived COVID-19 risk in Australia were controlled for. Interactions between health literacy and randomized conditions were also explored.

### Materials

Images and text for the intervention in phase 3 are provided in Multimedia Appendix 2. The health literacy–sensitive version of the text was developed by applying health literacy guidelines [18], using a web-based tool that provides objective feedback on the complexity of health information (eg, grade reading score, passive voice, and medical jargon) [19], and incorporating consumer feedback. We used the Sydney Health Literacy Lab Editor developed by our team [19] to meet the recommended grade 8 school level by simplifying complex words, sentences, and grammar. Participants randomized to the intervention were asked to choose their top 3 barriers to testing and then selected 1 barrier for the health literacy–sensitive action plan. This was adapted from our previous studies on health-related lifestyle changes to address intention-behavior gaps [20,21]. The web-based action plan used an *if-then* format (eg, “If I don’t want to get tested because there aren’t many cases in my area, then I will remind myself that every new outbreak of COVID-19 starts with one new case”), a format that has shown to improve various behavioral outcomes including smoking cessation, physical activity, and healthy eating [22-28]. Health literacy principles were applied to the if-then plan (eg, simple language, images, and breaking down tasks into smaller steps), as previous research has shown that this can improve the effectiveness of if-then plans for people with low health literacy [20,29]. The *if* options reflect the top 10 barriers for people with lower health literacy identified in phase 2:

- I would prefer to isolate instead.
- I’m not sure this symptom is one that needs testing.
- I have symptoms of COVID-19 but I don’t think they are bad enough.
- I have symptoms of COVID-19 but I think it’s a cold or hay fever.
- I’m worried the test is painful.
- I’m worried about spreading my illness on the way to the testing centre.
- There aren’t many cases in my area.
- I’m worried I will catch COVID-19 when I get tested or on the way to the testing centre.
- I’m not sure what to do.
- I’d like my doctor’s advice first.

Participants could then select a solution (*then* option). Solutions were generated in collaboration with our consumer representative.

Images and text for the phase 4 intervention are provided in Multimedia Appendix 3. There were 2 audio-visual interventions: one was an animation and the other was a TikTok-style video, which was more humorous. Both covered the following barriers to COVID-19 testing, which were identified as the most prevalent knowledge issues for people with lower health literacy in phase 2:

- I know what symptoms I have and don’t believe they are COVID-19 ones e.g. hay fever/normal cold.
- I’m not sure my symptoms are bad enough.
- It is unlikely I have COVID-19 because there aren’t many cases in my area.
- I’m not sure this symptom needs testing.

## Results

Participant characteristics for each phase are provided in Multimedia Appendix 4.

### Phase 1: Identification of COVID-19 Testing Barriers

Most people (1151/1369, 84.07%) agreed that they would get tested if they had COVID-19 symptoms (cough, fever, and sore throat), with 49.23% (674/1369) of people strongly agreeing. For self-isolation, 95.98% (1314/1369) of participants agreed to some extent that they would stay home if they had symptoms and 69.47% (951/1369) of participants strongly agreed. Most participants (982/1369, 71.73%) said they would get tested “no matter what.” The most common barriers selected from the list provided (Multimedia Appendix 1) were that testing is painful (153/1369, 11.18%), not knowing how to get tested (98/1369, 7.16%), and worry about getting infected at the testing center (81/1369, 5.92%). All other barriers were <3% (forgetting, worried what others think, too hard or expensive, doesn’t work or don’t trust results, and no one else getting tested). Many participants (136/1369, 9.93%) indicated other reasons, with 136 open responses that included many additional barriers to testing than those provided in the survey question. Table 2 maps all the barriers identified in open survey responses to the COM-B drivers of behavior (capability, opportunity, and motivation).

**Table 2:** Phase 1 COVID-19 testing behavior barriers mapped to the capability-opportunity-motivation-behavior (COM-B) model.

COM-B barriers			Example quotes from “other” barriers (10% of the sample)
**Physical capability**			
	My disability means I can’t get a test	“Fear of injury from getting tested—I have a deviated septum and narrow sinuses.*”*	
	I need a ramp/disability provisions for the testing centre	“Mobility and suppressed immune system make travel difficult.” “Testing facilities can’t accommodate my disability so it’s better for me just to stay home.*”*	
	I physically can’t access testing centre	“I’m homebound due to severe disability so I know arranging testing will be super hard.”	
**Psychological capability**			
	I have been getting conflicting information or being told not to get tested even with symptoms	“When I first got sore throat, headache, aches, cough, and partner very lethargic I got tested, and had the impression from testers that it wasn’t necessary with only those symptoms.”	
	I’m not sure how to get tested	“I dont know how to drive so I dont know how to get to places that test.”	
	I’m not sure my symptoms are bad enough	“Believing that I don’t meet the threshold eg when is a cough a cough or a runny nose with spicy food a runny nose? Otherwise I would get tested.”	
	I’m not sure this symptom is one that needs testing	“I am not sure whether I would be eligible to be tested.”	
**Physical opportunity**			
	The testing centres are hard to access—too far away from me	“Hard to get to where I can get tested.”	
	The opening hours of the testing centres don’t suit me	“Being able to get to the testing clinic within opening hours when caring for children.”	
	I don’t want to take public transport	“Public transport required to get to testing centre is inappropriate.”	
	I will need to take time off work	“hard to find the time with increased workload.”	
	I don’t have enough time to get tested	“Just tooooo busy—work during COVID.”	
	I don’t have childcare	“Depends on if I can get childcare and can find the time.”	
	If I get tested, it will impact me financially	“My husbands test took 10 days to get results. Which meant he could not work. He is self employed which means he also did not get paid and lost 8 days of income.”	
**Social opportunity**			
	I am worried what others will think of me having a test/being positive	“I will certainly feel worried about what others think of me, especially people who lives in the same household.”	
**Reflective motivation**			
	I had a bad experience when I got tested previously	“we had a bad experience getting my son tested...I can’t afford to be off work for 5 days just to chase down a negative result.”	
	I think the process of testing or pre-testing requirements is too much hassle/pointless	“There is a very tiny chance it will actually be COVID-19 so waste of time.”	
	I think the test is painful	“It hurt last time I got tested.”	
	I am worried about spreading my illness	“Have to ask someone to take me to a clinic and don’t want to get them sick.”	
	I’m worried I will catch covid myself whilst getting tested or en route	“I’m immunocompromised so don’t want to be around others in case I get sick.”	
	I don’t want to hear that I’m positive	“Will only get tested if I have a fever. Also, ‘too scared’ to know the results!”	
	I don’t think the testing works or results are reliable enough	“the accuracy of tests does concern me a bit.”	
	I don’t want to self-isolate after the test	“requirement to quarantine between test & getting results.”	
	I would prefer to self-isolate instead (or do another type of test)	“Don’t think it’s necessary to test, just isolate.”	
	Symptoms due to something else	“The symptoms are the same as my normal June sniffles I wouldn’t bother, unless I get other unusual symptoms.”	
	I have already got tested for these symptoms and was negative	“I was tested last week. If this residual cough remains I am unlikely to get retested. If I get new symptoms I would get retested.”	
	I have the symptoms but will wait for them to get worse first/threshold	“I’ll get tested if the symptoms last for more than 24 hours” “if its just something like runny nose but no other symptoms show up—No but if I have more than 1 then I’ll get tested.”	
	I don’t believe it can be covid as it’s so rare	“No community transmission, highly unlikely to be COVID-19.”	
	I will only get a test if advised to by my GP^a^	“Get tested if my dr refers me to be tested.”	
	I think getting tested may result in problems with my visa or job	“Might be excluded from starting a new job.”	
	Misinformation or myths/different views on managing COVID	“I believe in herd immunity.”	
**Automatic motivation**			
	I am scared of the test	“Afraid of the uncomfortable test.”	

^a^GP: general practitioner.

### Phase 2: Prevalence of Barriers to Testing for COVID-19

The aim of phase 2 was to estimate the prevalence of COVID-19 testing barriers in a nationally representative sample and explore health literacy disparities to identify priority issues for intervention.

Table 3 presents the prevalence and importance of barriers among the participants who selected any barrier (941/2034, 46.26%). The top barriers were related to motivation: “I know what symptoms I have and don’t believe they are COVID-19 ones e.g. hayfever/normal cold” (selected by 562/2034, 27.63%, with 200/2034, 9.83% ranking it most important) and “It is unlikely I have COVID-19 because there aren’t many cases in my area” (366/2034, 17.99% selected, with 104/2034, 5.11% ranking it most important). Capability issues were also common: “I’m not sure my symptoms are bad enough” (387/2034, 19.03% selected, with 109/2034, 5.36% ranking it most important) and “I’m not sure this symptom is one that needs testing” (306/2034, 15.04% selected, with 66/2034, 3.24% ranking it most important). Social opportunity issues were uncommon: 5.75% (117/2034) of participants were worried about what others might think if they got a positive COVID-19 test result, and 3.69% (75/2034) of participants worried about what others would think if they got tested at all. Physical opportunity issues included disabilities, access (especially the distance to travel to a testing center), and time restrictions.

When we compared participants with low health literacy versus high health literacy, we found similar results for the top 10 barriers, covering reflective motivation and psychological capability. However, there were significant differences between the 2 groups. People with low health literacy were more likely to select certain capability issues (I’m not sure how to get tested, *P*<.001; I’m not sure if this symptom needs testing, *P*=.01) and motivation issues (I would prefer to self-isolate instead, *P*<.001; I think the test is painful, *P*=.002; I’m worried about spreading my illness, *P*<.001, I’m worried I will catch COVID-19, *P*=.001; I don’t want to hear that I’m positive, *P*<.001; and I don’t trust people who are asking me to take a test, *P*=.02), compared with the people with higher health literacy (Table 3).

**Table 3:** Phase 2 prevalence of barriers in nationally representative sample and health literacy disparities (November 2020; n=2034).

Barrier^a^	Selections (n=2034), n (%)	People to rank it first (n=2034), n (%)	Low health literacy (n=334), n (%)	High health literacy (n=1681), n (%)	*P* value for health literacy
I would prefer to self-isolate instead	341 (16.8)	73 (3.6)	71 (21.3)	194 (11.5)	<.001
I know what symptoms I have and don’t believe they are COVID-19 ones e.g. hay fever/normal cold	562 (27.6)	200 (9.8)	61 (18.3)	241 (14.3)	.07
I think the test is painful	276 (13.6)	67 (3.3)	49 (14.7)	154 (9.2)	.002
I’m not sure my symptoms are bad enough	387 (19)	109 (5.4)	45 (13.5)	182 (10.8)	.16
I’m not sure this symptom is one that needs testing	306 (15)	66 (3.2)	41 (12.3)	134 (8)	.01
I am worried about spreading my illness on the way to the testing centre	207 (10.2)	39 (1.9)	39 (11.7)	105 (6.2)	<.001
It is unlikely I have COVID-19 because there aren’t many cases in my area	366 (18)	104 (5.1)	37 (11.1)	159 (9.5)	.36
I’m worried I will catch COVID-19 myself whilst getting tested or on the way to the testing centre	219 (10.8)	34 (1.7)	37 (11.1)	104 (6.2)	.001
I’m not sure how to get tested	123 (6)	27 (1.3)	36 (10.8)	59 (3.5)	<.001
I will only get tested if my GP^b^ tells me I should	187 (9.2)	63 (3.1)	30 (9.0)	126 (7.5)	.35
I don’t want to take public transport	143 (7)	31 (1.5)	24 (7.2)	80 (4.8)	.07
I have already got tested for these symptoms and was negative	150 (7.4)	34 (1.7)	24 (7.2)	71 (4.2)	.02
I think the process of testing is too much effort	148 (7.3)	15 (0.7)	24 (7.2)	78 (4.6)	.05
I don’t want to hear that I’m positive for COVID-19	85 (4.2)	5 (0.2)	21 (6.3)	42 (2.5)	<.001
I have been told not to get tested even if I have symptoms	113 (5.6)	33 (1.6)	18 (5.4)	65 (3.9)	.20
I don’t trust people who are asking me to take a test	72 (3.5)	16 (0.8)	17 (5.1)	45 (2.7)	.02
I am worried what others will think of me being positive for COVID-19	117 (5.8)	29 (1.4)	16 (4.8)	66 (3.9)	.47
I don’t want to take up resources for testing so that others can’t get tested	88 (4.3)	8 (0.4)	15 (4.5)	41 (2.4)	.04
I am worried what others will think of me having a test	75 (3.7)	6 (0.3)	14 (4.2)	43 (2.6)	.10
I don’t think the testing works or results are reliable enough	66 (3.2)	15 (0.7)	13 (3.9)	44 (2.6)	.20
I had a bad experience when I got tested before	89 (4.4)	10 (0.5)	12 (3.6)	49 (2.9)	.51
I don’t understand why I need to get tested	48 (2.4)	12 (0.6)	9 (2.7)	34 (2)	.44
I don’t want to self-isolate after the test	118 (5.8)	16 (0.8)	9 (2.7)	46 (2.7)	.97
I have the symptoms but will wait for them to get worse first	88 (4.3)	9 (0.4)	6 (1.8)	45 (2.7)	.35
I think getting tested may result in problems with my visa or with official bodies	33 (1.6)	5 (0.2)	6 (1.8)	21 (1.2)	.43

^a^Ordered from highest to lowest frequency in the low health literacy group (top 10 used in phase 3); participants could select more than 1 barrier.

^b^GP: general practitioner.

### Phase 3: Randomized Experiment to Test the Effect of Health Literacy–Sensitive Written Information About COVID-19 Testing Barriers

The aim of phase 3 was to address the top 10 testing barriers for people with lower health literacy. Opportunity barriers, such as physical inaccessibility, could not be addressed by a communication intervention, so this phase focused on capability and motivation issues. For the intervention, participants chose 3 relevant barriers from the 10 provided and viewed health literacy–sensitive versions of written government information about COVID-19 testing (eg, reading level reduced to grade 8 by replacing complex words and sentences with simpler options). Intervention participants then selected 1 barrier to make an “if-then” action plan for how they would get around this issue if they needed to get a COVID-19 test for symptoms. Immediately after the intervention, no differences were found for intervention versus control (written government frequently asked questions about COVID-19 testing) on intentions, knowledge, or any other psychological outcomes. After 4 weeks, 57.71% (790/1369) of respondents completed follow-up measures, but there were no differences between intervention and control (government tool that tailored information to the local context; eg, state-specific COVID-19 testing requirements). No significant differences were found in health literacy levels when included as an interaction term (Table 4).

**Table 4:** Phase 3 experiment results comparing intervention and control groups (February-March 2021; n=1314).

Outcomes^a^	Control (n=668), n (%)	Intervention (n=645), n (%)	Effect estimate (95% CI)	*P* value
Testing intentions (self—if symptomatic next 4 weeks; 7=strongest intentions)	5.8 (1.6)	5.8 (1.6)	0.07 (−0.09 to 0.22)	.40
Self-isolation intentions (7=strongest intentions)	6.2 (1.2)	6.1 (1.3)	−0.03 (−0.16 to 0.10)	.63
Knowledge total score (count/6)^b^	5.5 (0.9)	5.4 (1.0)	0.98 (0.94 to 1.03)	.42
Perceived risk (5=highest perceived risk)	2.8 (0.8)	2.8 (0.8)	−0.01 (−0.10 to 0.08)	.79
Threat to Australia (10=very serious threat)	6.7 (2.4)	6.7 (2.4)	0.04 (−0.21 to 0.29)	.77
Self-efficacy (7=highest self-efficacy)	5.8 (1.0)	5.7 (1.1)	0.00 (−0.11 to 0.11)	.96
COVID-19 stigma (7=highest stigma)	3.4 (1.2)	3.6 (1.3)	0.08 (−0.05 to 0.21)	.25
Distancing intentions (7=strongest intentions)	6.3 (1.1)	6.2 (1.2)	−0.03 (−0.15 to 0.08)	.56
Hand washing intentions (7=strongest intentions)	6.4 (1.0)	6.4 (1.1)	−0.04 (−0.14 to 0.07)	.49
Mask wearing intentions (7=strongest intentions)	6.0 (1.4)	6.0 (1.4)	0.02 (−0.13 to 0.16)	.81
Follow-up: Self-reported testing behavior^c^	39 (9.4)	39 (10.5)	1.00 (0.67 to 1.51)	.99
Follow-up: Testing intentions (7=strongest intentions)	5.8 (1.5)	5.7 (1.6)	−0.10 (−0.30 to 0.11)	.36
Follow-up: Testing intentions (controlling for baseline intentions) (7=strongest intentions)	—^d^	—	−0.11 (−0.30 to 0.08)	.26
Follow-up: Self-isolation intentions (7=strongest intentions)	6.2 (1.2)	6.1 (1.2)	−0.10 (−0.25 to 0.06)	.24
Follow-up: Self-isolation intentions (controlling for baseline intentions; 7=strongest intentions)	—	—	−0.05 (−0.20 to 0.09)	.47

^a^Continuous outcomes were analyzed using linear regression to estimate marginal mean differences.

^b^Count variables were analyzed using Poisson regression to estimate relative risks.

^c^Dichotomous outcomes were analyzed using generalized linear models with a modified Poisson approach (log link and robust SEs) to estimate relative risks.

^d^Not available.

### Phase 4: Randomized Experiment to Test the Effect of Health Literacy–Sensitive Audio-Visual Interventions About COVID-19 Testing Barriers in Adults With Lower Health Literacy

The aim of phase 4 was to develop a more targeted communication intervention with further refined testing of outcome measures. We selected 4 capability (knowledge) barriers from the most prevalent issues for people with lower health literacy in phase 2 and developed 2 audio-visual intervention scripts to address these: a simple animation in the style of Australian government advertisements and a TikTok-style video developed from the same information by a pharmacist with a large social media following for COVID-19 information videos. Immediately postintervention, we found that the animation and TikTok versions were more effective than the written government information for increasing knowledge about COVID-19 testing but not testing intentions or other psychological outcomes (based on a multiple-comparison adjusted α level of .025). No significant differences were found in health literacy levels when included as an interaction term (Table 5).

**Table 5:** Phase 4 experiment results comparing intervention and health literacy groups (November 2021; n=1527).

Outcome^a^	Government text (n=509)	Animation (n=514)	TikTok (n=504), n (%)	Animation vs government text		TikTok vs government text		Condition × health literacy interaction (*P* value)
				Effect estimate (95% CI)	*P* value	Effect estimate (95% CI)	*P* value	
Positive testing intentions (self—if symptoms tomorrow)^b^, n (%)	246 (48.3)	294 (57.2)	277 (55)	1.08 (0.99 to 1.17)	.08	1.10 (1.00 to 1.20)	.04	.70
Positive testing intentions (other—if symptoms tomorrow)^b^, n (%)	270 (53)	264 (51.4)	260 (51.6)	0.93 (0.83 to 1.03)	.17	0.95 (0.85 to 1.07)	.40	.31
Positive testing intentions (self—if symptoms next 4 weeks)^b^, n (%)	358 (70.3)	373 (72.6)	367 (72.8)	1.00 (0.93 to 1.07)	.97	1.02 (0.95 to 1.09)	.56	.24
Knowledge total score (count/6), median (IQR)^c^	3.0 (2.0 to 4.0)	4.0 (3.0 to 5.0)	4.0 (3.0 to 5.0)	1.33 (1.24 to 1.42)	<.001	1.25 (1.17 to 1.34)	<.001	.84
Self-efficacy, mean (SD)	5.6 (1.2)	5.7 (1.1)	5.7 (1.1)	0.07 (−0.05 to 0.20)	.26	0.13 (0.01 to 0.26)	.04	.13
Perceived effectiveness, mean (SD)	3.7 (0.8)	3.6 (1.0)	3.6 (0.9)	−0.09 (−0.17 to −0.01)	.04	−0.09 (−0.17 to 0.00)	.04	.84
Message credibility, mean (SD)	5.3 (1.4)	5.3 (1.5)	5.3 (1.4)	−0.04 (−0.16 to 0.08)	.53	−0.06 (−0.18 to 0.06)	.30	.20
Personal relevance, mean (SD)	4.2 (1.4)	4.2 (1.5)	4.1 (1.5)	−0.09 (−0.24 to 0.06)	.22	−0.13 (−0.28 to 0.02)	.09	.10
Other behavioral intentions (averaged: self-isolation, distancing, hand washing, and mask wearing), mean (SD)	5.7 (1.3)	5.8 (1.3)	5.8 (1.3)	0.08 (−0.05, 0.21)	.23	0.10 (−0.02, 0.23)	.12	.17

^a^Continuous outcomes were analyzed using linear regression to estimate marginal mean differences.

^b^Dichotomous outcomes were analyzed using generalized linear models with a modified Poisson approach (log link and robust SEs) to estimate relative risks.

^c^Count variables were analyzed using Poisson regression to estimate relative risks.

## Discussion

This study aimed to identify and address barriers to COVID-19 testing and test the effectiveness of multiple eHealth interventions on knowledge for people with varying health literacy levels.

### Principal Findings

Phase 1 identified a wide range of barriers to COVID-19 testing that had not been previously described in the COVID-19 literature. These covered all 3 behavioral drivers in the COM-B model. Phase 2 found that motivation and capability barriers were far more prevalent than opportunity barriers in Australia at the time of this study. Many barriers were reported as more prevalent among people with lower health literacy. Phases 3 and 4 tested different ways to address capability and motivation barriers. Phase 3 found no differences between standard government text about COVID-19 testing and a tailored text intervention that addressed many different barriers using health literacy design principles. Phase 4 found that audio-visual interventions to address key knowledge barriers are more effective than written government information for improving knowledge, but this was not enough to shift COVID-19 testing intentions in adjusted analyses.

### Comparison With Prior Work

Since our Australian research was conducted, new papers have been published on the issue of COVID-19 testing, including the move from PCR to rapid antigen tests (RATs). Survey, interview, and media analysis studies have identified similar capability, opportunity, and motivation barriers in other countries, including the United States, the United Kingdom, the United Arab Emirates, and Jordan [30-34], as well as in Australia (eg, in a recent qualitative study [35]). Sample populations have included the general public, parents, university students and staff, people experiencing homelessness, and specific cultural groups [36-44]. This study is unique in terms of mapping barriers to the COM-B theoretical framework, estimating the prevalence of key barriers to testing in people with diverse health literacy, and testing communication interventions to address capability and motivation barriers. We have shown that it is possible to increase COVID-19 testing knowledge when key barriers are explicitly addressed using simple audio-visual intervention formats that are cheap and quick to produce. However, it is important to acknowledge that a mass communication strategy will not address all barriers.

### Interpretation and Implications

The results of this study provide new insights into identifying and addressing behavioral barriers to COVID-19 testing, which is central to understanding and controlling COVID-19 and future pandemics. The large range of barriers identified in this study reflects the fact that COVID-19 testing was a complex new behavior at the time of the research, requiring a multifaceted approach to improve uptake depending on key barriers in different communities. Although the Australian PCR testing system was free and widely accessible for the first 2 years of the pandemic, the shift to using RATs introduced new opportunity barriers that were not an issue at the time of these studies. For example, it was very difficult to locate RATs during a period of low supply and high demand in January 2022, and there were issues with price gouging that made this unaffordable for many Australians [45]. Government regulation and funded RATs were subsequently introduced to limited groups, such as schoolchildren and pensioners [46,47].

### Future Research

The capability and motivation issues identified in this study apply to PCR testing, but there are likely to be additional barriers to RATs, which were not approved in Australia at the time of the study. Different barriers encountered for RATs are being investigated in subsequent research, including individuals’ ability to understand instructions, perform self-testing, and interpret the results correctly (eg, see trial ACTRN12622001517763). Concerns have been raised about the misinterpretation of negative results from RATs, which have a high error rate if the test is not used within the recommended period after exposure to a COVID-19 case [48]. Another avenue for further work is to partner with the media to avoid the identification and stigmatization of individuals with positive test results in future disease outbreaks [49]. Media reports and anecdotal data from frontline health professionals may be a useful way to quickly identify emerging local issues that could inform the measurement of testing barriers to make them more relevant to local communities.

### Strengths and Limitations

This research began as an unfunded and rapidly developing response to the COVID-19 pandemic. By addressing methodological issues and building on the findings in each phase, we were able to better target the final intervention and show the value of audio-visual formats in addressing common knowledge barriers among people with varying health literacy needs.

Phase 1 identified the range of barriers to COVID-19 testing in Australia for the first time, but the prevalence of the most important barriers could not be ascertained from these findings because of the reliance on open responses and a nonrepresentative sample. The next phase addressed these methodological issues. Phase 2 identified the prevalence of barriers to COVID-19 testing in a nationally representative sample and highlighted important health literacy disparities. However, even the second phase was not representative of all community groups, particularly those from culturally and linguistically diverse backgrounds, which has been identified as a key area of need in Australia and worldwide. We conducted a separate survey with these groups using interpreters to conduct the survey in phase 1 in preferred languages as a partnership with the Western Sydney Local Health District [50-52].

In phase 3, the intervention’s highly tailored design meant that there was considerable heterogeneity in the intervention elements that participants received. This may have contributed to the lack of observed effect. It is possible that there were ceiling effects for testing intentions when participants assumed that they would get tested when they were not currently or recently thinking about the logistics of getting tested. It is also possible that participants did not engage with the text-based intervention content. We attempted to address these methodological issues in the final phase 4 by focusing on a consistent set of key knowledge barriers in a more targeted group with lower baseline testing intentions (younger and lower education), using a more engaging intervention format (animation with text and audio and a social media–style video), and including more sensitive measures of testing intention to avoid potential biases.

The audio-visual interventions produced for phase 4 have information that is specific to the Australian context and may not be useful in other countries but can be used as a starting point for new knowledge interventions. We found that a simple and relatively cheap animation focused on key messages or a TikTok-style video that incorporated humor was both effective for increasing knowledge but not testing intentions. However, the findings may not be generalizable to other contexts, particularly where opportunity issues such as cost or physical access to testing are a problem. We expect that there will be additional barriers to RATs. Nevertheless, this study provides a comprehensive list of testing barriers that may help us better prepare for future variants or the next pandemic.

### Conclusions

To prepare for future pandemics, capability, opportunity, and motivation issues need to be addressed to increase testing behaviors for novel viruses, particularly in groups with lower health literacy. Audio-visual interventions can be used to address key knowledge issues in target populations. Our findings support broader advice from international experts on behavior change, highlighting the importance of diagnosing behavioral barriers to increase adherence to COVID-19 prevention behaviors.

## Appendix

Multimedia Appendix 1 Phase 1 and 2 questionnaires.

Multimedia Appendix 2 Screenshots from phase 3.

Multimedia Appendix 3 Phase 4 materials.

Multimedia Appendix 4 Participant characteristics.

Multimedia Appendix 5 CONSORT-eHEALTH checklist (V. 1.6.1).

## Abbreviations

COM-B: capability-opportunity-motivation-behavior
PCR: polymerase chain reaction
RAT: rapid antigen test

## Data Availability

Anonymous data are available from the authors.
