# Supplementary material for: Addressing Behavioral Barriers to COVID-19 Testing With Health Literacy–Sensitive eHealth Interventions: Results From 2 National Surveys and 2 Randomized Experiments
Source: JMIR Public Health Surveill. 2023 Jun 29;9:e40441. doi: 10.2196/40441 (PMC10337324; doi:10.2196/40441)
Supplement: Multimedia Appendix 1 [file publichealth_v9i1e40441_app1.docx]

**Covid-19 Survey Wave 3 (social media)**

1. How old are you?
2. What is your postcode?
3. Have you had a cough, sore throat, runny nose, cold or flu-like symptoms in the last 4 weeks?

Yes No Don't know

1. Do you know, or think, that you have COVID-19, or had it in the last 4 weeks?

Yes No Don't know

1. Do you know of anyone that has, or thinks they have, COVID-19 right now?

Yes No Don’t know

1. Do you know of anyone that had, or thinks they had, COVID-19 in the last 4 weeks?

Yes No Don’t know

1. Over the next 4 weeks, I plan to: (1=strongly disagree to 5=strongly agree)

- Get tested if I have COVID-19 symptoms (cough, sore throat, fever)
- Stay home if I have COVID-19 symptoms (cough, sore throat, fever)
- Wash my hands or use sanitiser to protect me and others from COVID-19
- Stay 1.5m away from others that I don't live with where I can

1. If I get COVID-19 symptoms in the next 4 weeks, the following might stop me from getting tested (select all the answers that apply):
   - I will get tested no matter what
   - I don't know how, when and where to get tested
   - Testing doesn't work / I don't trust the results
   - Testing is painful
   - I'm worried I will get infected with COVID-19 at the testing clinic
   - No one else is getting tested
   - I'm worried about what others think of me
   - It's too difficult or expensive to get tested
   - I'll forget to get tested
   - Other (please detail)
2. Please indicate how confident you are in the following: (1=Not confident at all to 5= extremely confident

- that you have the necessary information to make decisions about COVID-19?
- about your knowledge of COVID-19 management?
- about your knowledge of social distancing?

1. Over the last 4 weeks, COVID-19 has had a huge impact on my daily routine.

- Strongly disagree
- Disagree
- Disagree a bit
- Neither agree nor disagree
- Agree a bit
- Agree
- Strongly agree

1. Are there any people outside those in your household who you have seen regularly or had close contact with in the last 4 weeks? (Close contact is being within 1.5 metres of someone else for 15 minutes or longer, or being in the same closed space as someone else for 2 hours or longer)

Yes No

1. Please can you select the option that describes your relationship to these people:

- Close family member or relative
- Partner
- Friend
- Neighbour
- Work colleague
- I care for someone else outside of my home
- Other

1. Has there been any change in your employment status due to COVID-19 since the last survey (about 4 weeks ago)?

Yes No

1. Has there been any change in your employment status due to COVID-19 since the last survey?
2. What was the change?
   - Lost job
   - Stood down, not working for pay, but not fired
   - Pay cut
   - Reduction of hours
   - Not working but still being paid
   - Back in paid work
   - Increase in hours
   - Other (please detail)
3. Over the past week, how often have you felt nervous or "stressed" because of COVID-19?
   - Never
   - Some of the time
   - Most of the time
   - All of the time
4. Over the past week, how often have you felt alone or lonely because of COVID-19?
   - Never
   - Some of the time
   - Most of the time
   - All of the time
5. Which are your top 3 information sources about COVID-19 from the last 4 weeks?
   - Public TV (eg ABC, SBS)
   - Commercial TV (eg Nine, Seven, Ten)
   - Public Radio/Podcasts (eg ABC, Triple J, SBS)
   - Commercial Radio/Podcasts (eg Today FM, Nova)
   - Social Media
   - Newspaper/Magazines - online
   - Newspaper/Magazines - print
   - Government website
   - Other (non-government) website, NOT social media or newspapers
   - Your Doctor
   - Pharmacist, nurse or other health professional
   - Email notifications (e.g. from employees, services or subscriptions)
   - Family/friends
   - Other (please detail)
6. How long do you spend on average each day getting news or learning about COVID-19?
   - No time at all
   - Just a few minutes every day
   - Less than 30 minutes every day
   - Between 30 minutes and 1 hour every day
   - 1 to 2 hours every day
   - More than 2 hours every day
7. On a scale of 1 to 10, how serious of a public health threat do you think COVID-19 is currently?
8. On a scale of 1 to 10, how serious of a public health threat do you think COVID-19 is currently, in Australia?
9. On a scale of 1 to 10, how serious of a public health threat do you think COVID-19 is currently, globally?
10. Do you think that you will get sick from COVID-19?

- Not at all
- It's possible
- I probably will
- I definitely will

1. How likely do you think it is that you or someone you know will get sick from COVID-19 this year?
   - Not at all likely
   - Not that likely
   - Somewhat likely
   - Very likely
2. Since the COVID-19 restrictions started, have you had a telemedicine/telehealth appointment (appointment with your health provider by video or phone instead of an in-person appointment)?

Yes No Don’t know

1. Since the COVID-19 restrictions started, have you had a telemedicine/telehealth appointment
2. How many telehealth appointments have you had?
3. Since the COVID-19 restrictions started, have you had a telemedicine/telehealth appointment?

Yes

1. Were your telemedicine/telehealth visit(s) done by:
   - Telephone
   - Video conference (e.g. Zoom, Skype, Facebook video chat)
   - Both
2. If Since the COVID-19 restrictions started, have you had a telemedicine/telehealth appointment

Yes

1. How did your telemedicine/telehealth visit compare to a traditional in-person medical visit?

- Better than a traditional visit
- Just as good as a traditional visit
- Worse than a traditional visit
- Not sure

1. How did your telemedicine/telehealth visit compare to a traditional in-person medical visit?

Worse than a traditional visit?

1. How did your telemedicine/telehealth visit compare to a traditional in-person medical visit?

Not sure

1. Please tell us why:
2. Since the COVID-19 restrictions started, have you had a telemedicine/telehealth appointment?

Yes

1. How useful do you think it will be to have medical appointments with telemedicine/telehealth after the COVID-19 emergency is over?

- Extremely
- Very
- Moderately
- Slightly
- Not at all

1. Have you cancelled or postponed an appointment with a health professional in the last 4 weeks because of COVID-19?

Yes

No

1. Have you cancelled or postponed an appointment with a health professional in the last 4 weeks?

Yes

1. Why?
   - I was concerned about cost
   - I am isolating due to COVID-19 symptoms or risk
   - I was worried about travelling on public transport because of the COVID-19 risk
   - I did not want to go to a health or hospital clinic because of concerns about catching COVID-19 there
   - Too busy
   - Other (please tell us)
2. Did you feel you needed to see a health professional in person in the last 4 weeks but chose not to go?

Yes

No

1. Did you feel you needed to see a health professional in person in the last 4 weeks but chose not... = Yes

Why?

- I was concerned about cost
- I am isolating due to COVID-19 symptoms or risk
- I was worried about travelling on public transport because of the COVID-19 risk
- I did not want to go to a health or hospital clinic because of concerns about catching COVID-19 there
- Too busy
- Other (please tell us)

1. Have you needed to access a telehealth service in the last 4 weeks but could not?

Yes

No

1. Have you needed to access a telehealth service in the last 4 weeks but could not?
2. What was the main reason that you could not access a telehealth service in the last 4 weeks?

- Telehealth not available from GP or other health professional
- I do not have internet
- I am not able to use the internet
- Dislike or fear of the service
- Appointment not available when required
- Other (please detail)

1. How confident are you that your state or local government can prevent further spread of COVID-19?

- Not confident at all
- Not very confident
- Somewhat confident
- Very confident

1. How confident are you that the federal government can prevent further spread of COVID-19?

- Not confident at all
- Not very confident
- Somewhat confident
- Very confident

1. Please tell us how you have been feeling over the last 4 weeks. Mark how much you agree or disagree with the following statements: 1 = strongly disagree to 7= strongly agree

- I am confident in information about COVID-19 provided by the government (conf_gov3_1)
- I am satisfied with the amount of information about COVID-19 provided by the government (conf_gov3_2)
- I follow government advice on social distancing to help protect the wider community (conf_gov3_3)
- I am concerned that government recommendations about COVID-19 are not safe, or not enough is being done (conf_gov3_4)

1. Please mark how much you agree or disagree with the following statements: 1= strongly disagree to 5= strongly agree

- Much of the information we receive is wrong (1)
- I often disagree with commonly held views about the world (4)
- Official government accounts of events cannot be trusted (5)
- Major events are not always what they seem (6)

1. Please indicate how much you agree or disagree with these statements: 1 = strongly disagree to 7= strongly agree

- The threat of COVID-19 is greatly exaggerated (pseudo_2)
- The government restrictions are stronger than is needed (pseudo_4)

1. What restriction would be most important to you to loosen?
2. Please mark how much you agree or disagree with the following statements: 1 = definitely false to 5 = I don't know enough to make a decision

- 5G networks are spreading the virus (pseudo_wave3_01)
- Hot temperatures kill the virus (pseudo_wave3_02)
- Vitamin C is an effective treatment (pseudo_wave3_03)
- Ibuprofen exacerbates COVID-19 (pseudo_wave3_04)
- The flu shot provides immunity to COVID-19 (pseudo_wave3_05)
- Hydroxychloroquine is an effective treatment (pseudo_wave3_06)
- UV rays kill the virus (pseudo_wave3_07)
- There is a cure/vaccine for COVID-19 (pseudo_wave3_08)
- Parcels from China can spread the virus (pseudo_wave3_09)
- The COVID-19 virus was engineered and released from a Chinese laboratory in Wuhan (pseudo_wave3_10)

1. Please indicate how strongly you agree with the following statements, with 1 being do not agree at all and 10 being agree very strongly:

- My actions will influence whether or not I get COVID-19 (actions_1)
- It is my responsibility to follow all public health guidance to prevent the spread of COVID-19 to others (actions_2)
- Managing my health has become more difficult during the COVID-19 outbreak (actions_3)

1. Please indicate how much you agree or disagree with these statements: 1 = strongly disagree to 7= strongly agree

- Social distancing is important for my family's health (social_dist_1)
- Social distancing is important for the health of others in my community (social_dist_4)
- When everyone else is socially distancing, I don't need to (social_dist_5)
- I socially distance to protect people with a weaker immune system (social_dist_6)
- I would be willing to wear a face mask in crowded indoor spaces such as public transport or a shopping centre (social_dist_7)

1. How often did you leave your home over the last 4 weeks?

- Less than once per week
- Once per week
- A few times per week
- Once per day
- Multiple times per day

1. Please tell us what you have been doing over the last 4 weeks. Mark how much you agree or disagree with the following statements: 1= strongly disagree to 7= strongly agree

- I stay at home unless I need to shop for food or medicine, exercise, go to work, or provide care/support to another (1)
- I wash my hands frequently with soap and water (for at least 20 seconds). For example, before and after eating, after going to the toilet, and after going outside (2)
- I stay 1.5m away from other people outside my home (3)
- I avoid any exercise outside (6)
- I avoid close contact with anyone with cold or flu like symptoms (7)
- I avoid touching my eyes, nose and mouth with unwashed hands (8)
- I clean and disinfect frequently touched surfaces each day (e.g. phones, keyboards, door handles, light switches, bench tops) (9)
- I have stopped shaking hands, hugging or kissing as a greeting (10)

1. I have got the flu vaccine this year:

Yes

No

1. How likely is it that you will get the flu vaccine this year?

- Extremely unlikely
- Moderately unlikely
- Slightly unlikely
- Neither likely nor unlikely
- Slightly likely
- Moderately likely
- Extremely likely

1. If a COVID-19 vaccine becomes available, I will get it.

- Strongly disagree
- Disagree
- Somewhat disagree
- Neither agree nor disagree
- Somewhat agree
- Agree
- Strongly agree

1. Please explain your choice:
2. Have you downloaded the new COVID-19 tracing App from the Government (COVID Safe)?

Yes

No

1. I think it's safe that schools have re-opened for all students full time.

- Strongly disagree
- Disagree
- Somewhat disagree
- Neither agree nor disagree
- Somewhat agree
- Agree
- Strongly agree

1. The changes in the restrictions are difficult to keep up with.

- Strongly disagree
- Disagree
- Somewhat disagree
- Neither agree nor disagree
- Somewhat agree
- Agree
- Strongly agree

1. I think that a second wave of COVID-19 is likely to be a serious public health threat to Australia.

- Strongly disagree
- Disagree
- Somewhat disagree
- Neither agree nor disagree
- Somewhat agree
- Agree
- Strongly agree

1. A number of statements which people have used to describe themselves are given below. Please indicate how you feel right now, at this moment. There are no right or wrong answers, just give the answer which seems to describe your present feelings best. 1= not at all to 4 = very much

- I feel calm
- I am tense
- I feel upset
- I am relaxed
- I feel content
- I am worried

1. Please indicate how you have been feeling over the last 2 weeks: 1 = at no time to 6= all of the time

- I have felt cheerful and in good spirits
- I have felt calm and relaxed
- I have felt active and vigorous
- I woke up feeling fresh and rested
- My daily life has been filled with things that interest me

1. In your life, have you experienced any positive effects from the COVID-19 pandemic?

Yes No

1. In your life, have you experienced any positive effects from the COVID-19 pandemic?
2. Please describe what these positive experiences have been:
3. So that we can understand where there are areas of concern or confusion in the community, please tell us what information you would like to know or don't understand about COVID-19:
4. Do you have any questions or comments about what you think has caused COVID-19?

**Covid-19 Survey Wave 5 (6 months)**

1. How old are you?
2. What is your highest level of education?

- Less than Year 12 (less than high school)
- Year 12 (high school graduate)
- Certificate level I to IV
- Advanced Diploma and Diploma level
- Bachelor degree level and above

1. Would you like to receive feedback on this study?

- Yes
- No

1. What is your gender?

- Male
- Female
- Other/prefer not to say

1. Which state or territory do you live in?

- Australian Capital Territory
- Northern Territory
- New South Wales
- Victoria
- Queensland
- Western Australia
- South Australia
- Tasmania

1. What is your postcode?
2. Were you born in Australia?
3. Which country were you born in?
4. In which year did you arrive in Australia?
5. What is the main language you speak at home?

- English
- Other

1. What is the main language you speak at home?
2. Are you of Aboriginal or Torres Strait Islander origin?

- Yes
- No
- Not stated

1. Do you have private health insurance?

- Yes
- No
- Not stated

1. Has your doctor ever told you that you have: yes or no

- Respiratory disease
- Asthma
- COPD (chronic obstructive pulmonary disease)
- High blood pressure
- Cancer
- Heart disease
- Stroke
- Diabetes
- Depression
- Anxiety

1. About how many alcoholic drinks do you have each week?
2. Do you have any children?

- Yes, and I live with them
- Yes, but I don't live with them
- No

1. If Do you have any children? = Yes, and I live with them

Do you have any children? = Yes, but I don't live with them

How many children do you have?

- (1)
- (2)
- (3)
- (4)
- or more (5)

1. How confident are you with filling out medical forms by yourself?

- Not at all
- A little bit
- Somewhat
- Quite a bit
- Extremely

1. How good are you at working with fractions? 1= not good at all to 6= extremely good
2. How good are you at figuring out how much a shirt will cost if it is 20% off? 1= not good at al to 6= extremely good
3. How often do you find numerical information to be useful? 1 = never to 6= very often
4. Can you name 3 symptoms that are associated with COVID-19?
5. Can you name 3 steps people can take to prevent themselves or others from getting COVID-19?
6. This question may be difficult to think about or imagine, but what percent of people who get COVID-19 in Australia do you think will die as a result?
   Please provide a number (out of 100). No percentage (%) symbol is needed.
7. What percent of people who get COVID-19 do you think will only have mild symptoms?
   Please provide a number (out of 100).
8. Have you had any signs (symptoms) of COVID-19 in the last 4 weeks? (e.g. cough, cold, sore throat)

- Yes
- No

1. Do you know, or think, that you have COVID-19?

- Yes
- No
- Don't know

1. If Do you know, or think, that you have COVID-19? = Yes
2. Have you spoken to a healthcare professional about this?

- Yes
- No

1. If Have you spoken to a healthcare professional about this? = No
2. If you think you have been exposed to COVID-19 and/or you are experiencing symptoms, please call your doctor for medical advice.
3. If Do you know, or think, that you have COVID-19? = Yes
4. Or Have you had any signs (symptoms) of COVID-19 in the last 4 weeks? (e.g. cough, cold, sore throat) = Yes
5. Have you been tested for COVID-19?

- Yes
- In process
- Unable to get testing
- No

1. If Have you been tested for COVID-19? = Yes
2. What was the outcome of the testing?

- Positive
- Negative
- Pending

1. Do you know of anyone that has, or thinks they have, COVID-19?

- Yes
- No
- Don't know

1. If Do you know of anyone that has, or thinks they have, COVID-19? = Yes
2. How many people do you know who have, or think they have, COVID-19?

- Fewer than 5
- Between 5 and 10
- More than 10

1. Please indicate how confident you are in the following: 1= not confident at all to 5= extremely confident

- How confident are you that you have the necessary information to make decisions about COVID-19?
- How confident are you about your knowledge of COVID-19 management?
- How confident are you about your knowledge of social distancing?

1. How would you describe your current employment situation? Please choose all that apply:

- Working now for pay
- Unemployed
- Retired
- Student
- Unable to work due to disability or illness
- Volunteer
- Stay at home parent/caregiver
- Other

1. If How would you describe your current employment situation? Please choose all that apply: = Other

Please detail:

1. If How would you describe your current employment situation? Please choose all that apply: = Working now for pay
2. Are you working from home or outside the home?

- From home
- Outside the home
- Other

If How would you describe your current employment situation? Please choose all that apply: = Working now for pay

Or How would you describe your current employment situation? Please choose all that apply: = Unemployed

1. What type of work do you do?
2. Has there been any change in your employment status due to COVID-19 in the last 4 weeks?

- Yes
- No

1. What was the change?

- Lost job
- Stood down, not working for pay, but not fired
- Pay cut
- Reduction of hours
- Not working but still being paid
- Back in paid work
- Increase in hours
- Other (please detail)

1. Please indicate how you feel about the following statements: 1= not at all to 5 = very much

- I worry about the financial problems I will have in the future as a result of the COVID-19 pandemic
- I am satisfied with my current financial situation
- I am able to meet my weekly expenses
- I feel financially stressed
- I am concerned about keeping my job and income, including work at home
- The COVID-19 pandemic has reduced my satisfaction with my present financial situation

1. Over the past week, how often have you felt nervous or "stressed" because of COVID-19?

- Never
- Some of the time
- Most of the time
- All of the time

1. Over the past week, how often have you felt alone or lonely because of COVID-19?

- Never
- Some of the time
- Most of the time
- All of the time

1. Where have you been getting information about COVID-19? Pick up to 3 information sources.

- Public TV (eg ABC, SBS)
- Commercial TV (eg Nine, Seven, Ten)
- Public Radio/Podcasts (eg ABC, Triple J, SBS)
- Commercial Radio/Podcasts (eg Today FM, Nova)
- Social Media
- Newspaper/Magazines - online
- Newspaper/Magazines - print
- Government website
- Other (non-government) website, NOT social media or newspapers
- Your Doctor
- Pharmacist, nurse or other health professional
- Email notifications (e.g. from employees, services or subscriptions)
- Family/friends
- Other (please detail below):

If Where have you been getting information about COVID-19? Pick up to 3 information sources. = Social Media

1. Which social media channels have you been getting information about COVID-19 from?

- YouTube
- Facebook
- Instagram
- Twitter
- TikTok
- Twitch
- Weibo
- Wechat
- Other (please specify below)

1. How long do you spend on average each day getting news or learning about COVID-19?

- No time at all
- Just a few minutes every day
- Less than 30 minutes every day
- Between 30 minutes and 1 hour every day
- 1 to 2 hours every day
- More than 2 hours every day

1. On a scale of 1 to 10, with 1 being not at all difficult and 10 being extremely difficult:

- How difficult has it been for you to find accurate, understandable information about COVID-19?
- Has government advice been difficult to understand?

1. Please provide reasons for your answers above:
2. On a scale of 1 to 10, how serious of a public threat do you think COVID-19 is currently?
3. On a scale of 1 to 10, how serious of a public threat do you think COVID-19 is currently, in Australia?
4. On a scale of 1 to 10, how serious of a public threat do you think COVID-19 is currently, globally?
5. If Do you know, or think, that you have COVID-19? = No
6. Or Do you know, or think, that you have COVID-19? = Don't know
7. Do you think that you will get sick from COVID-19?

- Not at all
- It's possible
- I probably will
- I definitely will

1. How likely do you think it is that you or someone you know will get sick from COVID-19 this year?

- Not at all likely
- Not that likely
- Somewhat likely
- Very likely

1. How confident are you that your state or local government can prevent further outbreak of COVID-19?

- Not confident at all
- Not very confident
- Somewhat confident
- Very confident

1. How confident are you that the federal government can prevent further outbreak of COVID-19?

- Not confident at all
- Not very confident
- Somewhat confident
- Very confident

1. Please indicate how much you agree with the following: 1= strongly disagree to 7 = strongly agree

- I am confident in information about COVID-19 provided by the government
- I am satisfied with the amount of information about COVID-19 provided by the government
- I follow government advice on social distancing to help protect the wider community
- I am concerned that government recommendations about COVID-19 are not safe, or not enough is being done

1. Please indicate how much you trust the following, with 1 being do not trust at all and 7 being trust very much:

- Scientists involved in developing and testing new ways to control COVID-19
- Researchers involved in tracking and predicting COVID-19 cases
- Medical institutions (GPs, hospitals) involved in managing COVID-19 cases

1. Please indicate how much you agree or disagree with these statements: 1= strongly disagree to 7= strongly agree

- Data about the effectiveness of vaccines is often made up
- The threat of COVID-19 is greatly exaggerated
- Herd immunity would be beneficial for managing COVID-19 and this fact is covered up
- The government restrictions are stronger than is needed

1. Please mark how much you agree or disagree with the following statements: 1 = definitely false to 5 = I don’t know enough to make a decision

- 5G networks are spreading the virus
- Vitamin C is an effective treatment
- Ibuprofen exacerbates COVID-19
- The flu shot provides immunity to COVID-19
- Hydroxychloroquine is an effective treatment
- There is a cure/vaccine for COVID-19 right now
- Parcels from China can spread the virus
- The COVID-19 virus was engineered and released from a Chinese laboratory in Wuhan
- Information about treatments for COVID-19 is being suppressed by those who want the pandemic to continue
- COVID-19 is not more dangerous than seasonal flu
- If people wear masks it will slow the development of widespread immunity to COVID-19
- If people social distance it will slow the development of widespread immunity to COVID-19
- Countries with hot weather have no reported cases of COVID-19
- COVID-19 can be transmitted in areas with hot and humid climates
- People of all ages can be infected with COVID-19

1. Please indicate how strongly you agree with the following statements, with 1 being do not agree at all and 10 being agree very strongly:

- My actions will influence whether or not I get COVID-19
- It is my responsibility to follow all public health guidance to prevent the spread of COVID-19 to others
- Managing my health has become more difficult during the COVID-19 outbreak

1. Please indicate how much you agree or disagree with these statements: 1 = strongly disagree to 7 = strongly agree

- Social distancing is important for my family's health
- Social distancing is important for the health of others in my community
- When everyone else is socially distancing, I don't need to
- I socially distance to protect people with a weaker immune system

1. Please indicate how much you agree or disagree with these phrases: 1 = strongly disagee to 7 = strongly agree

- I stay at home unless I need to shop for food or medicine, exercise, go to work, or provide care/support to another
- I wash my hands frequently with soap and water (for at least 20 seconds). For example, before and after eating, after going to the toilet, and after going outside
- I stay 1.5m away from other people outside my home
- I avoid any exercise outside
- I avoid close contact with anyone with cold or flu like symptoms
- I avoid touching my eyes, nose and mouth with unwashed hands
- I clean and disinfect frequently touched surfaces each day (e.g. phones, keyboards, door handles, light switches, bench tops)
- I have stopped shaking hands, hugging or kissing as a greeting
- I avoid small gatherings in enclosed spaces e.g. family celebrations

1. I got tested if I had COVID-19 symptoms (cough, sore throat, fever) in the last 4 weeks

- Yes
- No
- I haven't had symptoms

1. I stayed at home if I had COVID-19 symptoms (cough, sore throat, fever) in the last 4 weeks

- Strongly disagree
- Disagree
- Somewhat disagree
- Neither agree nor disagree
- Somewhat agree
- Agree
- Strongly agree
- N/A

1. I have got the flu vaccine this year:

- Yes
- No

1. Please explain your choice:
2. If I have got the flu vaccine this year: = No
3. How likely is it that you will get the flu vaccine this year?

- Extremely unlikely
- Moderately unlikely
- Slightly unlikely
- Neither likely nor unlikely
- Slightly likely
- Moderately likely
- Extremely likely

1. If a COVID-19 vaccine becomes available, I will get it.

- Strongly disagree
- Disagree
- Somewhat disagree
- Neither agree nor disagree
- Somewhat agree
- Agree
- Strongly agree

1. Please explain your choice:
2. Over the next 4 weeks, I plan to: strongly disagree = 1 to strongly agree = 7

- Get tested if I have COVID-19 symptoms (cough, sore throat, fever)
- Stay home if I have COVID-19 symptoms (cough, sore throat, fever)
- Wash my hands or use sanitiser to protect me and others from COVID-19
- Stay 1.5m away from others that I don't live with where I can

The next few pages will ask you about different reasons why people may not get a test for COVID-19. 
Please select the reasons that apply to you. You don't have to pick a reason on each page.
If you have already had signs of COVID-19 but decided not to get tested, think back to your reasons for this.

1. Below are some of the reasons why people don't want to get tested, or can't get tested, for COVID-19. Please select the reasons that apply to you. Pick as many or as few as you want.
   If I get COVID-19 symptoms (signs of having COVID-19), I might not get tested because...

- I have been told not to get tested even if I have symptoms
- I'm not sure how to get tested
- I'm not sure my symptoms are bad enough
- I'm not sure this symptom is one that needs testing
- I don't understand why I need to get tested
- I don't want to take public transport
- I am worried what others will think of me having a test
- I am worried what others will think of me being positive for COVID-19
- None of these apply to me

1. Below are some more of the reasons we've found for why people don't want to get tested, or can't get tested, for COVID-19. Again, please select the reasons that apply to you. 
   If I get COVID-19 symptoms (signs of having COVID-19), I might not get tested because...

- I had a bad experience when I got tested before
- I think the process of testing is too much effort
- I think the test is painful
- I am worried about spreading my illness on the way to the testing centre
- I'm worried I will catch COVID-19 myself whilst getting tested or on the way to the testing centre
- I don't want to hear that I'm positive for COVID-19
- I don't think the testing works or results are reliable enough
- I don't want to self-isolate after the test
- None of these apply to me

1. Here is the final page of reasons for why people don't want to get tested, or can't get tested, for COVID-19. Again, please select the reasons that apply to you. 
   If I get COVID-19 symptoms (signs of having COVID-19), I might not get tested because...

- I don't trust people who are asking me to take a test
- I would prefer to self-isolate instead
- I know what symptoms I have and don't believe they are COVID-19 ones e.g. hayfever/normal cold
- I have already got tested for these symptoms and was negative
- I have the symptoms but will wait for them to get worse first
- It is unlikely I have COVID-19 because there aren't many cases in my area
- I don't want to take up resources for testing so that others can't get tested
- I will only get tested if my GP tells me I should
- I think getting tested may result in problems with my visa or with official bodies
- None of these apply to me

1. Now please re-order the answers you gave to the last question, from the most important reason at the top to the least important. Don't worry about it being exactly right, this will just help us understand broadly which reasons are more important to people.
   Drag the answers into your preferred order. 
   If there is nothing displayed below, just click the 'next' arrow to move on with the survey.

Display This Choice:

If Below are some of the reasons why people don't want to get tested, or can't get tested, for COVID... = I have been told not to get tested even if I have symptoms

______ I have been told not to get tested even if I have symptoms (1)

Display This Choice:

If Below are some of the reasons why people don't want to get tested, or can't get tested, for COVID... = I'm not sure how to get tested

______ I'm not sure how to get tested (2)

Display This Choice:

If Below are some of the reasons why people don't want to get tested, or can't get tested, for COVID... = I'm not sure my symptoms are bad enough

______ I'm not sure my symptoms are bad enough (3)

Display This Choice:

If Below are some of the reasons why people don't want to get tested, or can't get tested, for COVID... = I'm not sure this symptom is one that needs testing

______ I'm not sure this symptom is one that needs testing (4)

Display This Choice:

If Below are some of the reasons why people don't want to get tested, or can't get tested, for COVID... = I don't understand why I need to get tested

______ I don't understand why I need to get tested (5)

Display This Choice:

If Below are some of the reasons why people don't want to get tested, or can't get tested, for COVID... = I don't want to take public transport

______ I don't want to take public transport (6)

Display This Choice:

If Below are some of the reasons why people don't want to get tested, or can't get tested, for COVID... = I am worried what others will think of me having a test

______ I am worried what others will think of me having a test (7)

Display This Choice:

If Below are some of the reasons why people don't want to get tested, or can't get tested, for COVID... = I am worried what others will think of me being positive for COVID-19

______ I am worried what others will think of me being positive for COVID-19 (8)

Display This Choice:

If Below are some more of the reasons we've found for why people don't want to get tested, or can't... = I had a bad experience when I got tested before

______ I had a bad experience when I got tested before (10)

Display This Choice:

If Below are some more of the reasons we've found for why people don't want to get tested, or can't... = I think the process of testing is too much effort

______ I think the process of testing is too much effort (11)

Display This Choice:

If Below are some more of the reasons we've found for why people don't want to get tested, or can't... = I think the test is painful

______ I think the test is painful (12)

Display This Choice:

If Below are some more of the reasons we've found for why people don't want to get tested, or can't... = I am worried about spreading my illness on the way to the testing centre

______ I am worried about spreading my illness on the way to the testing centre (13)

Display This Choice:

If Below are some more of the reasons we've found for why people don't want to get tested, or can't... = I'm worried I will catch COVID-19 myself whilst getting tested or on the way to the testing centre

______ I'm worried I will catch COVID-19 myself whilst getting tested or on the way to the testing centre (14)

Display This Choice:

If Below are some more of the reasons we've found for why people don't want to get tested, or can't... = I don't want to hear that I'm positive for COVID-19

______ I don't want to hear that I'm positive for COVID-19 (15)

Display This Choice:

If Below are some more of the reasons we've found for why people don't want to get tested, or can't... = I don't think the testing works or results are reliable enough

______ I don't think the testing works or results are reliable enough (16)

Display This Choice:

If Below are some more of the reasons we've found for why people don't want to get tested, or can't... = I don't want to self-isolate after the test

______ I don't want to self-isolate after the test (17)

Display This Choice:

If Here is the final page of reasons for why people don't want to get tested, or can't get tested, f... = I don't trust people who are asking me to take a test

______ I don't trust people who are asking me to take a test (19)

Display This Choice:

If Here is the final page of reasons for why people don't want to get tested, or can't get tested, f... = I would prefer to self-isolate instead

______ I would prefer to self-isolate instead (20)

Display This Choice:

If Here is the final page of reasons for why people don't want to get tested, or can't get tested, f... = I know what symptoms I have and don't believe they are COVID-19 ones e.g. hayfever/normal cold

______ I know what symptoms I have and don't believe they are COVID-19 ones e.g. hayfever/normal cold (21)

Display This Choice:

If Here is the final page of reasons for why people don't want to get tested, or can't get tested, f... = I have already got tested for these symptoms and was negative

______ I have already got tested for these symptoms and was negative (22)

Display This Choice:

If Here is the final page of reasons for why people don't want to get tested, or can't get tested, f... = I have the symptoms but will wait for them to get worse first

______ I have the symptoms but will wait for them to get worse first (23)

Display This Choice:

If Here is the final page of reasons for why people don't want to get tested, or can't get tested, f... = It is unlikely I have COVID-19 because there aren't many cases in my area

______ It is unlikely I have COVID-19 because there aren't many cases in my area (24)

Display This Choice:

If Here is the final page of reasons for why people don't want to get tested, or can't get tested, f... = I don't want to take up resources for testing so that others can't get tested

______ I don't want to take up resources for testing so that others can't get tested (25)

Display This Choice:

If Here is the final page of reasons for why people don't want to get tested, or can't get tested, f... = I will only get tested if my GP tells me I should

______ I will only get tested if my GP tells me I should (26)

Display This Choice:

If Here is the final page of reasons for why people don't want to get tested, or can't get tested, f... = I think getting tested may result in problems with my visa or with official bodies

______ I think getting tested may result in problems with my visa or with official bodies (27)

1. Are there any other reasons we've missed that are important to you?

- Yes
- No

1. If Are there any other reasons we've missed that are important to you? = Yes

Are they listed below? Select all those that apply to you.

- My disability means I can't get tested
- I need a ramp or other provisions for the testing centre
- I physically can't access a testing centre
- The testing centres are too far away from me
- The opening hours of the testing centres don't suit me
- I've been given the wrong information about testing centres
- I will need to take time off work
- I don't have enough time to get tested
- I don't have childcare
- If I get tested, it will impact me financially
- I am scared of the test
- None of these apply to me

If Are there any other reasons we've missed that are important to you? = Yes

1. Are there any other reasons that you wouldn't want to get tested for COVID-19? Please tell us below:
2. Please indicate how much you agree or disagree with these statements: 1 = strongly disagree to 7= strongly agree

- In general, most of my close friends have similar beliefs about COVID-19 as me
- In general, my family has similar beliefs about COVID-19 as me

1. In general, would you say your health is:

- Poor
- Fair
- Good
- Very good
- Excellent

The next questions are about finding health information on the internet.

1. Please indicate whether you agree or disagree with the following: 1= strongly disagree to 5= strongly agree

- I know what health resources are available on the internet
- I know where to find helpful health resources on the Internet
- I know how to find helpful health resources on the Internet
- I know how to use the Internet to answer my questions about health
- I know how to use the health information I find on the Internet to help me
- I have the skills I need to evaluate the health resources I find on the Internet
- I can tell high quality health resources from low quality health resources on the Internet
- I feel confident in using information from the Internet to make health decisions

1. Please read each statement and choose a number 0, 1, 2 or 3 which indicates how much the statement applied to you over the past week. There are no right or wrong answers. Do not spend too much time on any statement.
   0 = Did not apply to me at all1 = Applied to me to some degree, or some of the time2 = Applied to me a considerable degree, or a good part of the time3 = Applied to me very much, or most of the time

- I was aware of dryness of my mouth
- I experienced breathing difficulty (e.g. excessively rapid breathing, breathlessness in the absence of physical exertion)
- I experienced trembling (e.g. in the hands)
- I was worried about situations in which I might panic and make a fool of myself
- I felt close to panic
- I was aware of the action of my heart in the absence of physical exertion (eg, sense of heart rate increase, heart missing a beat)
- I felt scared without any good reason

1. Please indicate how you have been feeling over the last 2 weeks: 1 = at no time to 6= all of the time

- I have felt cheerful and in good spirits
- I have felt calm and relaxed
- I have felt active and vigorous
- I woke up feeling fresh and rested
- My daily life has been filled with things that interest me

1. So that we can understand where there are areas of concern or confusion in the community, please tell us what information you would like to know or don't understand about COVID-19:
