# Supplementary material for: Addressing Behavioral Barriers to COVID-19 Testing With Health Literacy–Sensitive eHealth Interventions: Results From 2 National Surveys and 2 Randomized Experiments
Source: JMIR Public Health Surveill. 2023 Jun 29;9:e40441. doi: 10.2196/40441 (PMC10337324; doi:10.2196/40441)
Supplement: Multimedia Appendix 2 [file publichealth_v9i1e40441_app2.pdf]

Below are ten reasons why people may **not** want to get tested for COVID-19. Pick the 3 that are most relevant for you, then **click on them** to see more information.

If I get COVID-19 symptoms (signs of having COVID-19), I might not get tested because...

I would prefer to self-isolate instead

I'm not sure this sign is one that needs testing

I have signs of COVID-19 but I don't think they are bad enough

I have signs of COVID-19 but I think it's a cold or hay fever

I'm worried the test is painful

Im worried about spreading my illness on the way to the testing centre

There aren't many cases in my area.

I'm worried I will catch COVID-19 when I get tested or on the way to the testing centre

I'm not sure what to do

I'd like my doctor's advice first

Below are ten reasons why people may **not** want to get tested for COVID-19. Pick the 3 that are most relevant for you, then **click on them** to see more information.

If I get COVID-19 symptoms (signs of having COVID-19), I might not get tested because...

- I would prefer to self-isolate instead
- I'm not sure this sign is one that needs testing
- I have signs of COVID-19 but I don't think they are bad enough
- I have signs of COVID-19 but I think it's a cold or hay fever
- I'm worried the test is painful

Testing for COVID-19 can be uncomfortable but shouldn't be painful. The test itself is very quick, and usually takes less than one minute.

- Im worried about spreading my illness on the way to the testing centre
- There aren't many cases in my area.
- I'm worried I will catch COVID-19 when I get tested or on the way to the testing centre
- I'm not sure what to do
- I'd like my doctor's advice first

Below are ten reasons why people may not want to get tested for COVID-19. Pick the 3 that are most relevant for you, then **click on them** to see more information.

If I get COVID-19 symptoms (signs of having COVID-19), I might not get tested because...

I would prefer to self-isolate instead

I'm not sure this sign is one that needs testing

I have signs of COVID-19 but I don't think they are bad enough

I have signs of COVID-19 but I think it's a cold or hay fever

I'm worried the test is painful

Im worried about spreading my illness on the way to the testing centre

There aren't many cases in my area.

Every new outbreak of COVID-19 starts with one new case. It is much easier to stop the spread when cases are low because we can use testing to quickly find other people who have COVID-19, even people who don’t show any signs of the virus.

I'm worried I will catch COVID-19 when I get tested or on the way to the testing centre

I'm not sure what to do

I'd like my doctor's advice first

Getting tested

Getting tested for COVID-19 is important because it will reduce the spread of infection and keep us all safe

The next stage of this survey asks you to create an action plan for getting tested for COVID-19

→

COVID-19 Testing Plan

We want you to plan how you will get tested for COVID-19, even if there are obstacles in your way.

Step 1: Reasons you might hesitate to get tested

Below is a list of reasons why you might hesitate to get tested for COVID-19, even if you have signs of COVID-19.

Choose **up to 3** reasons from the list.

The most likely reasons I won't get tested for COVID-19 are...

The most likely reasons I won't get tested for COVID-19 are...

I would prefer to self-isolate instead	I am worried about spreading my illness on the way to the testing centre
I have signs of COVID-19 but I think it's a cold or hay fever	there aren't many cases in my area
I'm worried the test is painful	I'm worried I will catch COVID-19 when I get tested or on the way to the testing centre
I have signs of COVID-19 but I don't think they are bad enough	I'm not sure what to do
I'm not sure this sign is one that needs testing	

Step 2: Your 'key' reason

Below are your top 3 reasons to not get tested for COVID-19.

Some reasons will be more important than others. Choose the 1 that you feel is the **most likely reason** that you wouldn't get tested if you have signs of COVID-19.

I might not get tested for COVID-19 because...

I'm worried the test is painful	there aren't many cases in my area
I have signs of COVID-19 but I don't think they are bad enough	

Step 3: Make a plan

Great! Your reason is: Not getting tested when **there aren't many cases in my area**

The last step is to come up with a plan!

Choose one solution that you think will work best for you.

Drag it into the space on the right.

Items	If I don't want to get tested because there aren't many cases in my area, I will...
remind myself that even if I only have one of the signs, it is still worth getting tested	1 remind myself that every new outbreak of COVID-19 starts with one new case
remind myself that getting tested helps protect my community	
remind myself that getting tested means we can check if people I've been in contact with have COVID-19	

Step 4: Your plan is almost ready!

Here is your plan so far:

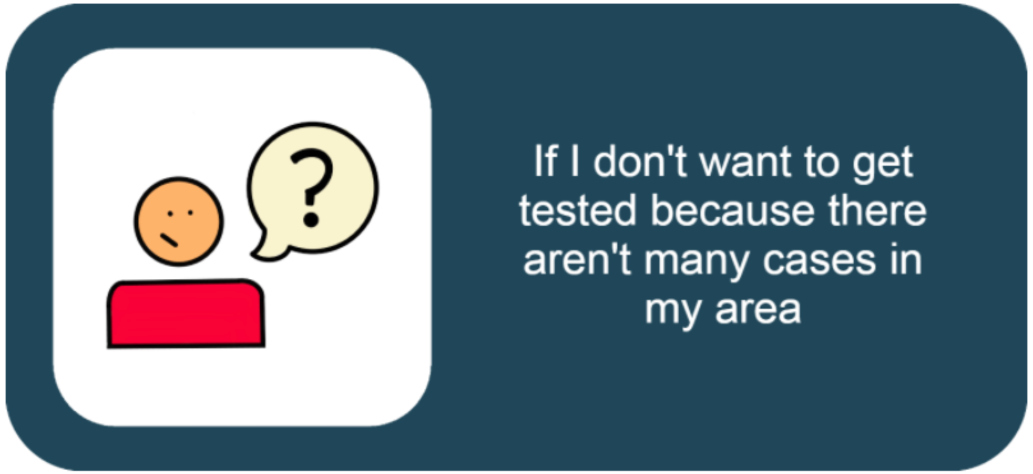

If I don't want to get tested because there aren't many cases in my area

Then I will remind myself that every new outbreak of COVID-19 starts with one new case

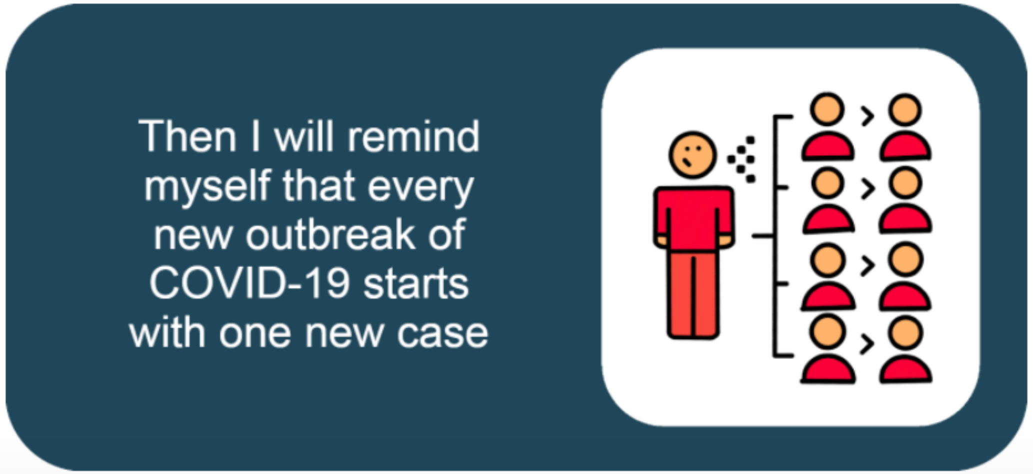

How hard do you think it will be to do this plan for the next month?

Very easy										Very hard
0	1	2	3	4	5	6	7	8	9	10

Your score suggests this plan may be too hard for you.

Let's choose something a little easier.

Choose the solution that you think will work best for you.

Drag it into the space on the right.

Items	If I don't want to get tested because there aren't many cases in my area, I will...
remind myself that even if I only have one of the signs, it is still worth getting tested	1 remind myself that getting tested helps protect my community
remind myself that every new outbreak of COVID-19 starts with one new case	
remind myself that getting tested means we can check if people I've been in contact with have COVID-19	

Your COVID-19 testing plan

Well done! Try to remember this plan for the **next month**. Say it **3 times** to yourself. You will be able to continue the survey in 10 seconds.

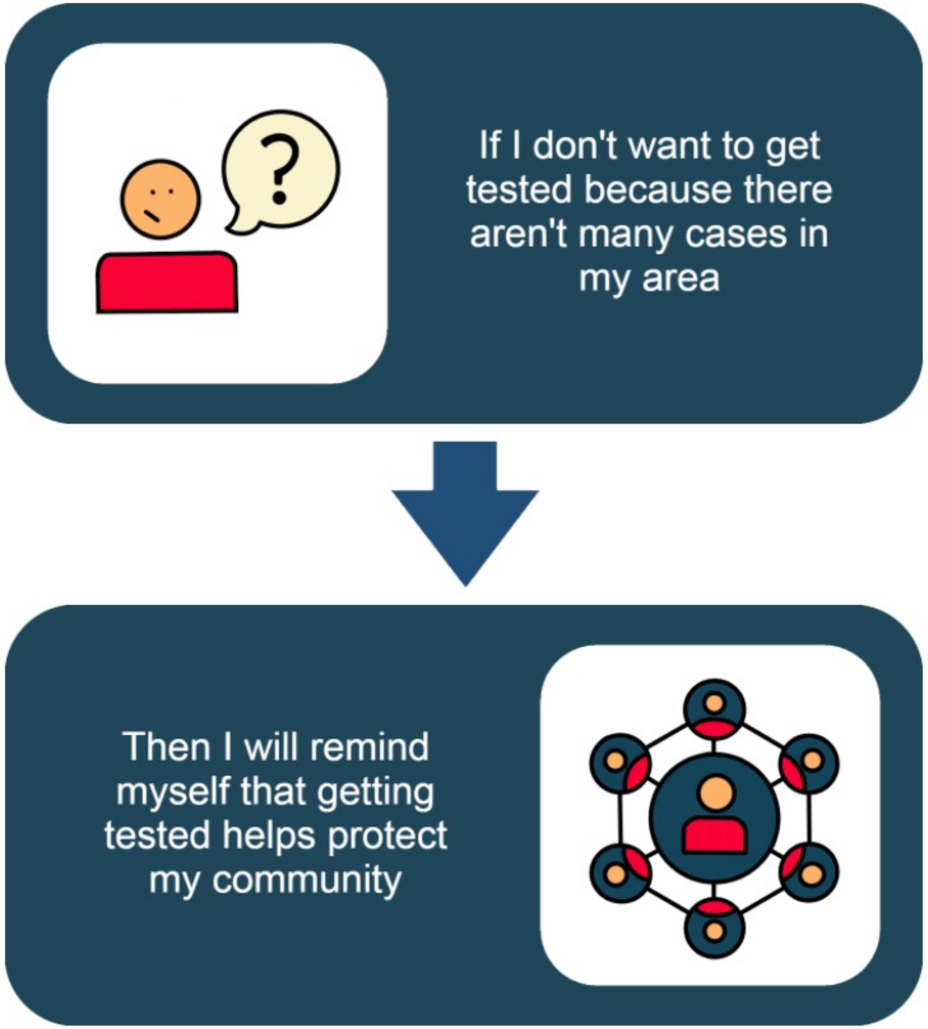

We will email you **three reminders** about your plan over the next four weeks.

You could also **print** a copy or take a **screenshot**. To download a PDF select 'Print as PDF' when you choose a printer.

Print or download plan
