# Supplementary material for: Addressing Behavioral Barriers to COVID-19 Testing With Health Literacy–Sensitive eHealth Interventions: Results From 2 National Surveys and 2 Randomized Experiments
Source: JMIR Public Health Surveill. 2023 Jun 29;9:e40441. doi: 10.2196/40441 (PMC10337324; doi:10.2196/40441)

**Phase 4 materials**

**Animation video:** <https://www.youtube.com/watch?v=Ygtf-YmyJVE>

**Animation video transcript:**

It’s easy to think that you don’t have to get tested for symptoms of COVID-19. Waking up with a sore throat is easy to write off as just a cold or nothing serious. But the symptoms of COVID-19 could be easily mistaken for a cold or hayfever, so it’s important to still get tested. Even if you only have one cold-like symptom, and it’s not that bad, you still need to get tested straight away. There may not have been many cases in your area, which is great. But this can change at any time. Every case needs to be found so that we can quickly let everyone in the community know what they need to do to limit the spread. COVID-19 vaccines work really well. They stop people from getting very sick from COVID. But it’s still important to get tested, even if you’ve already had the vaccine. Remember, every new outbreak of COVID-19 starts with one new case. You should get tested straight away for any of the following symptoms: fever, sore throat, cough, shortness of breath, runny nose, loss of taste or smell. Even if the symptoms are mild or haven’t lasted very long. And even if you’ve had the COVID-19 vaccine.

**Example animation video screenshots:**

**TikTok video:** <https://www.youtube.com/watch?v=rAMmPcoTkgk>

**TikTok video transcript:**

So let’s talk about COVID testing. One of the reasons why the COVID cases aren’t coming down is because people aren’t getting tested soon enough. And it kind of makes sense, I mean sometimes you might wake up and not have any symptoms at all. Or you might have symptoms and think it’s the common cold or hay fever. But early signs and symptoms of COVID might not be very specific. Sometimes it might just start out with symptoms similar to the common cold, like a sore throat or a runny nose. But that’s why it’s so important to get tested early anyway, even with mild symptoms. Now, while it is true that your state might not have many cases, which is fantastic, if you want to avoid ending up like some states, every single case needs to be found, and it needs to be found early. Finding cases early will allow us to let everyone in the community know and therefore allow us to track cases quicker to prevent an outbreak. Therefore, we won’t end up like some other states. Sometimes, even if you’re vaccinated, you can still catch COVID and pass it on. Since the vaccine is still protecting you from COVID you might not present with as many symptoms. Therefore, you should still get tested. So, what symptoms should you be looking out for? You should get tested straight away if you have symptoms of fever, sore throat, cough, shortness of breath, runny nose and loss of taste and smell. You should still get tested even if the symptoms are mild and short lived, and if you’re vaccinated. And don’t forget, every single outbreak starts with one case.

**Example TikTok video screenshots:**


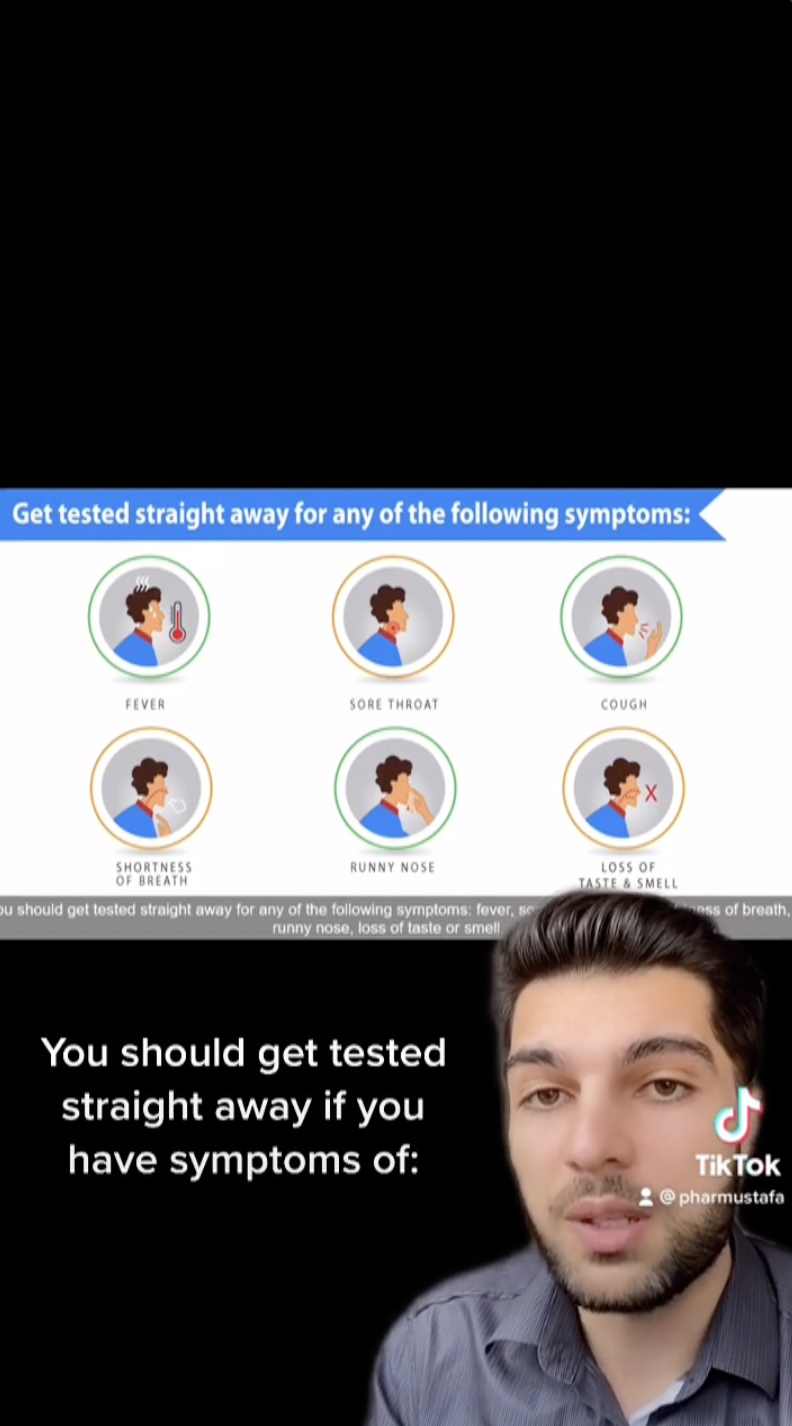


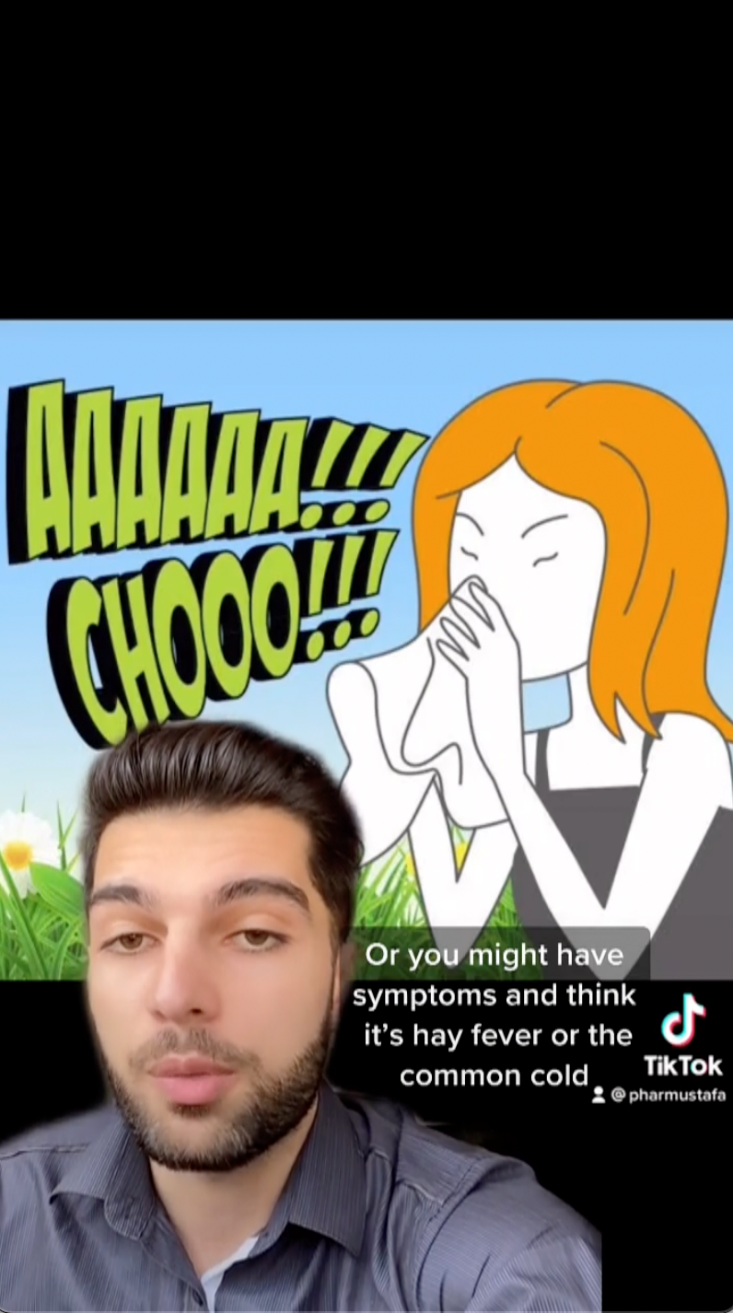


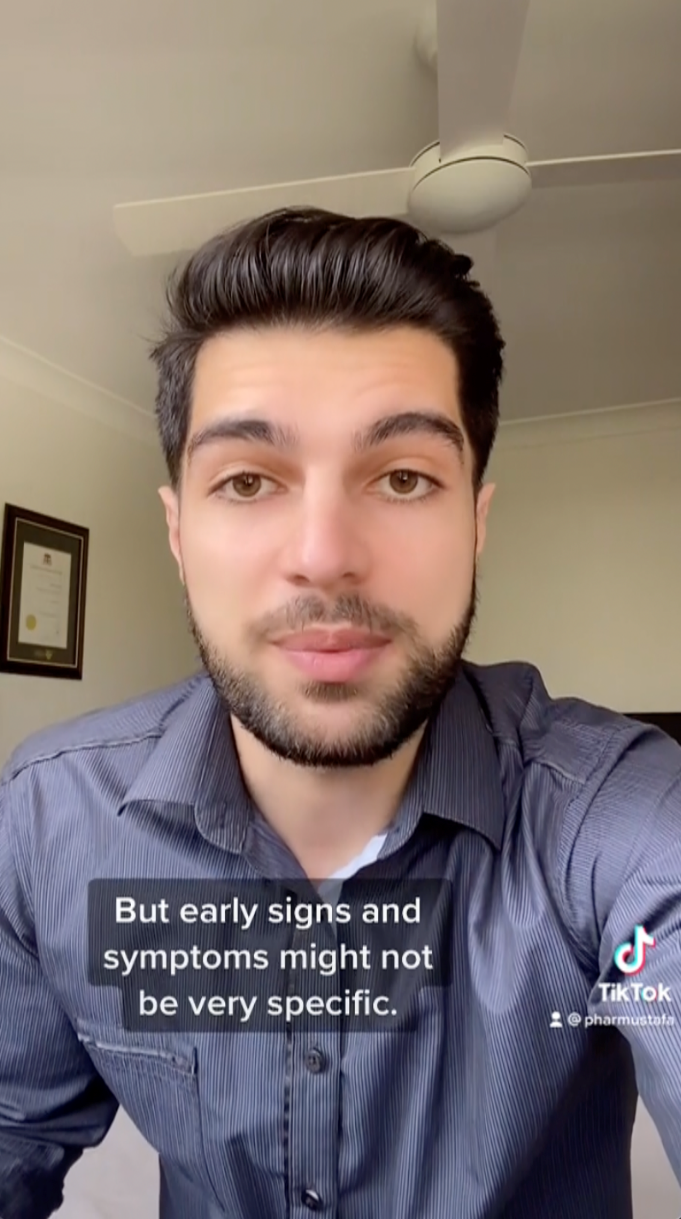

Supplement: Multimedia Appendix 3 [file publichealth_v9i1e40441_app3.docx]
