# Supplementary material for: Addressing Behavioral Barriers to COVID-19 Testing With Health Literacy–Sensitive eHealth Interventions: Results From 2 National Surveys and 2 Randomized Experiments
Source: JMIR Public Health Surveill. 2023 Jun 29;9:e40441. doi: 10.2196/40441 (PMC10337324; doi:10.2196/40441)
Supplement: Multimedia Appendix 4 [file publichealth_v9i1e40441_app4.docx]

### Participants

The sample demographics for each phase are shown in Tables 1 and 2. Phase 1 had under-representation of male gender (32%) and lower education (14.5%), with a relatively small sample for low health literacy (8.9%). Quota sampling in Phase 2 resulted in a more representative sample for male gender (49.5% male) and lower education (34.6%), which increased the sample for low health literacy (16.5%). Phase 3 recruited a representative age/gender sample in the state of New South Wales so we could use specific written government information as the control, as different states had different COVID-19 contexts at that time (e.g. different public communication about restrictions based on local case numbers). Phase 4 targeted a national sample of younger adults <40 years with quota sampling to ensure an adequate sample with lower education, where we only recruited from states that did not have specific testing requirements in place for local outbreaks (e.g. in hotspot areas of Sydney during Delta outbreak).

*Table 1: Participant characteristics for survey studies 1 and 2.*

| **Variable** | **Phase 1**  **(N=1369)** | **Phase 2**  **(n=2034)** |
| --- | --- | --- |
| *Age (years), mean (SD)* | 44.7 (16.7) | 45.6 (16.8) |
| *Age group, n (%)* |  |  |
| 18 to 25 years | 232 (16.9%) | 282 (13.9%) |
| 26 to 40 years | 372 (27.2%) | 625 (30.7%) |
| 41 to 55 years | 344 (25.1%) | 502 (24.7%) |
| 56 to 90 years | 421 (30.8%) | 625 (30.7%) |
| *Gender, n (%)* |  |  |
| Male | 433 (31.6%) | 1006 (49.5%) |
| Female | 911 (66.5%) | 1024 (50.3%) |
| Other / prefer not to say | 25 (1.8%) | 4 (0.2%) |
| *Highest level of educational attainment, n (%)* |  |  |
| High school or less | 198 (14.5%) | 704 (34.6%) |
| Certificate I-IV | 140 (10.2%) | 733 (36.0%) |
| University education | 1031 (75.3%) | 597 (29.4%) |
| *Health literacy, n (%)* |  |  |
| Adequate (high) | 1170 (91.1%) | 1699 (83.5%) |
| Inadequate (low) | 199 (8.9%) | 335 (16.5%) |
| *State, n (%)* |  |  |
| Australian Capital Territory | 48 (3.5%) | 41 (2.0%) |
| New South Wales | 721 (52.7%) | 642 (31.6%) |
| Northern Territory | 5 (0.4%) | 19 (0.9%) |
| Queensland | 183 (13.4%) | 422 (20.7%) |
| South Australia | 63 (4.6%) | 140 (6.9%) |
| Tasmania | 55 (4.0%) | 46 (2.3%) |
| Victoria | 205 (15.0%) | 511 (25.1%) |
| Western Australia | 90 (6.6%) | 213 (10.5%) |
| *Region, n (%)* |  |  |
| Rural | 342 (25.0%) | 591 (29.1%) |
| Metropolitan | 1027 (75.0%) | 1443 (70.9%) |
|  |  |  |

*Table 2: Participant characteristics for trial studies 3 and 4*

|  | **Phase 3 baseline** | | **Phase 3 follow-up** | | **Phase 4 baseline** | | |
| --- | --- | --- | --- | --- | --- | --- | --- |
| **Outcome** | **Control -**  **Gov FAQ text (n=668)** | **Intervention - Top barrier text (n=646)** | **Control - Gov info tool (n=414)** | **Intervention - Action plan tool (n=376)** | **Control -**  **Gov FAQ text (n=509)** | **Intervention - Animation (n=514)** | **Intervention - TikTok (n=504)** |
| Mean age | 47.4 | 46.9 | 52.1 | 53.7 | 31.1 | 31.3 | 30.9 |
| Gender splits | 295 (44%) male, 368 (55%) female, 5 (1%) non-binary/gender fluid | 292 (45%) male, 353 (55%) female, 1 (0.2%) non-binary/gender fluid | 191 (46.1%) male, 223 (53.9%) female | 199 (52.9%) male, 177 (47.1%) female | 142 (27.9%) male, 358 (70.3%) female, 6 (1.2%) non-binary/gender fluid, 2 (0.4%) other, 1 missing | 154 (30.0%) male, 347 (67.5%) female, 11 (2.1%) non-binary/gender fluid, 2 missing | 135 (26.8%) male, 359 (71.2%) female, 7 (1.4%) non-binary/gender fluid, 2 (0.4%) other, 1 missing |
| Education | 517 (77%) less than university | 508 (79%) less than university | 315 (76.1%) less than university | 293 (77.9%) less than university | 215 (42.2%) less than university | 217 (42.2%) less than university | 213 (42.3%) less than university |
| Health literacy | 103 (15%) inadequate HL | 100 (15%) inadequate HL | 56 (13.5%) inadequate HL | 47 (12.5%) inadequate HL | 80 (15.7%) inadequate HL | 82 (16.0%) inadequate HL | 82 (16.3%) inadequate HL |
| Aboriginal | 13 (2%) Aboriginal or Torres Strait Islander | 11 (2%) Aboriginal or Torres Strait Islander | 9 (2.2%) Aboriginal or Torres Strait Islander | 4 (1.1%) Aboriginal or Torres Strait Islander | 26 (5.1%) Aboriginal or Torres Strait Islander | 19 (3.7%) Aboriginal or Torres Strait Islander | 25 (5.0%) Aboriginal or Torres Strait Islander |
| Language | 38 (6%) non-English language | 40 (6%) non-English language | 21 (5.1%) non-English language | 19 (5.1%) non-English language | 40 (7.9%) non-English language | 34 (6.6%) non-English language | 40 (7.9%) non-English language |
| Mean trust (7=trust very much) | 5.2 | 5.2 | 5.3 | 5.3 | 4.9 | 4.9 | 5 |
| Living alone | 126 (19%) live alone | 99 (15%) live alone | 89 (21.5%) live alone | 57 (15.2%) live alone | Not measured | Not measured | Not measured |
| Prior covid test | 212 (32%) had had a prior covid test | 188 (29%) had had a prior covid test | 109 (26.3%) had had a prior covid test | 92 (24.5%) had had a prior covid test | 311 (61.1%) had had a prior covid test | 312 (60.7%) had had a prior covid test | 304 (60.3%) had had a prior covid test |
