# Supplementary material for: Addressing Behavioral Barriers to COVID-19 Testing With Health Literacy–Sensitive eHealth Interventions: Results From 2 National Surveys and 2 Randomized Experiments
Source: JMIR Public Health Surveill. 2023 Jun 29;9:e40441. doi: 10.2196/40441 (PMC10337324; doi:10.2196/40441)
Supplement: Multimedia Appendix 5 [file publichealth_v9i1e40441_app5.pdf]

CONSORT-EHEALTH (V 1.6.1) - Submission/Publication Form

The CONSORT-EHEALTH checklist is intended for authors of randomized trials evaluating web-based and Internet-based applications/interventions, including mobile interventions, electronic games (incl multiplayer games), social media, certain telehealth applications, and other interactive and/or networked electronic applications. Some of the items (e.g. all subitems under item 5 - description of the intervention) may also be applicable for other study designs.

The goal of the CONSORT EHEALTH checklist and guideline is to be
a) a guide for reporting for authors of RCTs,
b) to form a basis for appraisal of an ehealth trial (in terms of validity)

CONSORT-EHEALTH items/subitems are MANDATORY reporting items for studies published in the Journal of Medical Internet Research and other journals / scientific societies endorsing the checklist.

Items numbered 1., 2., 3., 4a., 4b etc are original CONSORT or CONSORT-NPT (non-pharmacologic treatment) items.
Items with Roman numerals (i., ii, iii, iv etc.) are CONSORT-EHEALTH extensions/clarifications.

As the CONSORT-EHEALTH checklist is still considered in a formative stage, we would ask that you also RATE ON A SCALE OF 1-5 how important/useful you feel each item is FOR THE PURPOSE OF THE CHECKLIST and reporting guideline (optional).

Mandatory reporting items are marked with a red *.
In the textboxes, either copy & paste the relevant sections from your manuscript into this form - please include any quotes from your manuscript in QUOTATION MARKS,
or answer directly by providing additional information not in the manuscript, or elaborating on why the item was not relevant for this study.

YOUR ANSWERS WILL BE PUBLISHED AS A SUPPLEMENTARY FILE TO YOUR PUBLICATION IN JMIR AND ARE CONSIDERED PART OF YOUR PUBLICATION (IF ACCEPTED).
Please fill in these questions diligently. Information will not be copyedited, so please use proper spelling and grammar, use correct capitalization, and avoid abbreviations.

DO NOT FORGET TO SAVE AS PDF _AND_ CLICK THE SUBMIT BUTTON SO YOUR ANSWERS ARE IN OUR DATABASE !!!

Citation Suggestion (if you append the pdf as Appendix we suggest to cite this paper in the caption):

Eysenbach G, CONSORT-EHEALTH Group
CONSORT-EHEALTH: Improving and Standardizing Evaluation Reports of Web-based and Mobile Health Interventions
J Med Internet Res 2011;13(4):e126
URL: http://www.jmir.org/2011/4/e126/
doi: 10.2196/jmir.1923
PMID: 22209829

carissabonner@gmail.com Switch account

Draft saved

Not shared

* Indicates required question

Your name *

First Last

Carissa Bonner

Primary Affiliation (short), City, Country *

University of Toronto, Toronto, Canada

University of Sydney, Australia

Your e-mail address *

abc@gmail.com

carissa.bonner@sydney.edu.au

Title of your manuscript *

Provide the (draft) title of your manuscript.

Behavioural barriers to COVID-19 testing: developing and testing health literacy interventions

Name of your App/Software/Intervention *

If there is a short and a long/alternate name, write the short name first and add the long name in brackets.

COVID-19 PCR testing videos

Evaluated Version (if any)

e.g. "V1", "Release 2017-03-01", "Version 2.0.27913"

Your answer

Language(s) *

What language is the intervention/app in? If multiple languages are available, separate by comma (e.g. "English, French")

English

URL of your Intervention Website or App

e.g. a direct link to the mobile app on app in appstore (itunes, Google Play), or URL of the website. If the intervention is a DVD or hardware, you can also link to an Amazon page.

https://www.youtube.com/watch?v=Ygtf-YmyJVE

URL of an image/screenshot (optional)

Your answer

Accessibility *

Can an enduser access the intervention presently?

- (●) access is free and open
- ( ) access only for special usergroups, not open
- ( ) access is open to everyone, but requires payment/subscription/in-app purchases
- ( ) app/intervention no longer accessible
- ( ) Other:

Primary Medical Indication/Disease/Condition *

e.g. "Stress", "Diabetes", or define the target group in brackets after the condition, e.g. "Autism (Parents of children with)", "Alzheimers (Informal Caregivers of)"

COVID-19

Primary Outcomes measured in trial *

comma-separated list of primary outcomes reported in the trial

Knowledge, intentions

Secondary/other outcomes

Are there any other outcomes the intervention is expected to affect?

Perceived credibility, emotional response, personal relevance

Recommended "Dose" *

What do the instructions for users say on how often the app should be used?

- ( ) Approximately Daily
- ( ) Approximately Weekly
- ( ) Approximately Monthly
- ( ) Approximately Yearly
- (x) "as needed"
- ( ) Other:

Approx. Percentage of Users (starters) still using the app as recommended after 3 months *

- (selected) unknown / not evaluated
- 0-10%
- 11-20%
- 21-30%
- 31-40%
- 41-50%
- 51-60%
- 61-70%
- 71%-80%
- 81-90%
- 91-100%
- Other:

Overall, was the app/intervention effective? *

- ( ) yes: all primary outcomes were significantly better in intervention group vs control
- (•) partly: SOME primary outcomes were significantly better in intervention group vs control
- ( ) no statistically significant difference between control and intervention
- ( ) potentially harmful: control was significantly better than intervention in one or more outcomes
- ( ) inconclusive: more research is needed
- ( ) Other:

Article Preparation Status/Stage *

At which stage in your article preparation are you currently (at the time you fill in this form)

- ( ) not submitted yet - in early draft status
- ( ) not submitted yet - in late draft status, just before submission
- ( ) submitted to a journal but not reviewed yet
- ( ) submitted to a journal and after receiving initial reviewer comments
- (•) submitted to a journal and accepted, but not published yet
- ( ) published
- ( ) Other:

Journal *

If you already know where you will submit this paper (or if it is already submitted), please provide the journal name (if it is not JMIR, provide the journal name under "other")

- ( ) not submitted yet / unclear where I will submit this
- ( ) Journal of Medical Internet Research (JMIR)
- ( ) JMIR mHealth and UHealth
- ( ) JMIR Serious Games
- ( ) JMIR Mental Health
- (•) JMIR Public Health
- ( ) JMIR Formative Research
- ( ) Other JMIR sister journal
- ( ) Other:

Is this a full powered effectiveness trial or a pilot/feasibility trial? *

- ( ) Pilot/feasibility
- (•) Fully powered

Manuscript tracking number *

If this is a JMIR submission, please provide the manuscript tracking number under "other" (The ms tracking number can be found in the submission acknowledgement email, or when you login as author in JMIR. If the paper is already published in JMIR, then the ms tracking number is the four-digit number at the end of the DOI, to be found at the bottom of each published article in JMIR)

- ○ no ms number (yet) / not (yet) submitted to / published in JMIR
- ◉ Other: 46785

TITLE AND ABSTRACT

1a) TITLE: Identification as a randomized trial in the title

1a) Does your paper address CONSORT item 1a? *

I.e does the title contain the phrase "Randomized Controlled Trial"? (if not, explain the reason under "other")

- ○ yes
- ◉ Other: Multi-phase project reported in the paper, so not limited to RCT

1a-i) Identify the mode of delivery in the title

Identify the mode of delivery. Preferably use "web-based" and/or "mobile" and/or "electronic game" in the title. Avoid ambiguous terms like "online", "virtual", "interactive". Use "Internet-based" only if Intervention includes non-web-based Internet components (e.g. email), use "computer-based" or "electronic" only if offline products are used. Use "virtual" only in the context of "virtual reality" (3-D worlds). Use "online" only in the context of "online support groups". Complement or substitute product names with broader terms for the class of products (such as "mobile" or "smart phone" instead of "iphone"), especially if the application runs on different platforms.

	1	2	3	4	5	
subitem not at all important	(●)	( )	( )	( )	( )	essential

Clear selection

Does your paper address subitem 1a-i? *

Copy and paste relevant sections from manuscript title (include quotes in quotation marks "like this" to indicate direct quotes from your manuscript), or elaborate on this item by providing additional information not in the ms, or briefly explain why the item is not applicable/relevant for your study

Multi-phase project reported in the paper, so not limited to web-based intervention

1a-ii) Non-web-based components or important co-interventions in title

Mention non-web-based components or important co-interventions in title, if any (e.g., "with telephone support").

	1	2	3	4	5	
subitem not at all important	(●)	( )	( )	( )	( )	essential

Clear selection

Does your paper address subitem 1a-ii?

Copy and paste relevant sections from manuscript title (include quotes in quotation marks "like this" to indicate direct quotes from your manuscript), or elaborate on this item by providing additional information not in the ms, or briefly explain why the item is not applicable/relevant for your study

Multi-phase project reported in the paper, so not limited to intervention but also surveys

1a-iii) Primary condition or target group in the title

Mention primary condition or target group in the title, if any (e.g., “for children with Type I Diabetes”) Example: A Web-based and Mobile Intervention with Telephone Support for Children with Type I Diabetes: Randomized Controlled Trial

	1	2	3	4	5	
subitem not at all important	○	○	○	◉	○	essential

Clear selection

Does your paper address subitem 1a-iii? *

Copy and paste relevant sections from manuscript title (include quotes in quotation marks "like this" to indicate direct quotes from your manuscript), or elaborate on this item by providing additional information not in the ms, or briefly explain why the item is not applicable/relevant for your study

"Behavioural barriers to COVID-19 testing"

1b) ABSTRACT: Structured summary of trial design, methods, results, and conclusions

NPT extension: Description of experimental treatment, comparator, care providers, centers, and blinding status.

1b-i) Key features/functionalities/components of the intervention and comparator in the METHODS section of the ABSTRACT

Mention key features/functionalities/components of the intervention and comparator in the abstract. If possible, also mention theories and principles used for designing the site. Keep in mind the needs of systematic reviewers and indexers by including important synonyms. (Note: Only report in the abstract what the main paper is reporting. If this information is missing from the main body of text, consider adding it)

	1	2	3	4	5	
subitem not at all important	○	○	○	○	◉	essential

Clear selection

Does your paper address subitem 1b-i? *

Copy and paste relevant sections from the manuscript abstract (include quotes in quotation marks "like this" to indicate direct quotes from your manuscript), or elaborate on this item by providing additional information not in the ms, or briefly explain why the item is not applicable/relevant for your study

"The intervention was developed through 4 phases. Phase 1 was a national survey in June 2020 (n=1369), where testing barriers were elicited and coded using the COM-B framework (capability-opportunity-motivation-behavior). Phase 2 was a national survey in November 2020 (n=2869) to estimate prevalence of testing barriers and health literacy disparities. Phase 3 was a randomised experiment testing health literacy-sensitive written information for a wide range of capability/motivation barriers in February-March 2021 (n=1314), where participants chose their top 3 barriers to testing to view a tailored intervention. Phase 4 was a randomised experiment testing 2 audio-visual interventions addressing common testing barriers for people with lower health literacy in November 2021, targeting young adults as a key group endorsing misinformation (n=1527)."

1b-ii) Level of human involvement in the METHODS section of the ABSTRACT

Clarify the level of human involvement in the abstract, e.g., use phrases like “fully automated” vs. “therapist/nurse/care provider/physician-assisted” (mention number and expertise of providers involved, if any). (Note: Only report in the abstract what the main paper is reporting. If this information is missing from the main body of text, consider adding it)

	1	2	3	4	5	
subitem not at all important	◉	○	○	○	○	essential

Clear selection

Does your paper address subitem 1b-ii?

Copy and paste relevant sections from the manuscript abstract (include quotes in quotation marks "like this" to indicate direct quotes from your manuscript), or elaborate on this item by providing additional information not in the ms, or briefly explain why the item is not applicable/relevant for your study

"Phase 3 was a randomised experiment testing health literacy-sensitive written information for a wide range of capability/motivation barriers in February-March 2021 (n=1314), where participants chose their top 3 barriers to testing to view a tailored intervention. Phase 4 was a randomised experiment testing 2 audio-visual interventions addressing common testing barriers for people with lower health literacy in November 2021, targeting young adults as a key group endorsing misinfotmation (n=1527)."

1b-iii) Open vs. closed, web-based (self-assessment) vs. face-to-face assessments in the METHODS section of the ABSTRACT

Mention how participants were recruited (online vs. offline), e.g., from an open access website or from a clinic or a closed online user group (closed usergroup trial), and clarify if this was a purely web-based trial, or there were face-to-face components (as part of the intervention or for assessment). Clearly say if outcomes were self-assessed through questionnaires (as common in web-based trials). Note: In traditional offline trials, an open trial (open-label trial) is a type of clinical trial in which both the researchers and participants know which treatment is being administered. To avoid confusion, use "blinded" or "unblinded" to indicated the level of blinding instead of "open", as "open" in web-based trials usually refers to "open access" (i.e. participants can self-enrol). (Note: Only report in the abstract what the main paper is reporting. If this information is missing from the main body of text, consider adding it)

	1	2	3	4	5	
subitem not at all important	○	○	◉	○	○	essential

Clear selection

Does your paper address subitem 1b-iii?

Copy and paste relevant sections from the manuscript abstract (include quotes in quotation marks "like this" to indicate direct quotes from your manuscript), or elaborate on this item by providing additional information not in the ms, or briefly explain why the item is not applicable/relevant for your study

Recruitment varied for each phase; as 4 phases are reported in abstract there is not space but this is detailed in methods section.

1b-iv) RESULTS section in abstract must contain use data

Report number of participants enrolled/assessed in each group, the use/uptake of the intervention (e.g., attrition/adherence metrics, use over time, number of logins etc.), in addition to primary/secondary outcomes. (Note: Only report in the abstract what the main paper is reporting. If this information is missing from the main body of text, consider adding it)

	1	2	3	4	5	
subitem not at all important	○	○	○	○	◉	essential

Clear selection

Does your paper address subitem 1b-iv?

Copy and paste relevant sections from the manuscript abstract (include quotes in quotation marks "like this" to indicate direct quotes from your manuscript), or elaborate on this item by providing additional information not in the ms, or briefly explain why the item is not applicable/relevant for your study

"The intervention was developed through 4 phases. Phase 1 was a national survey in June 2020 (n=1369), where testing barriers were elicited and coded using the COM-B framework (capability-opportunity-motivation-behavior). Phase 2 was a national survey in November 2020 (n=2869) to estimate prevalence of testing barriers and health literacy disparities. Phase 3 was a randomised experiment testing health literacy-sensitive written information for a wide range of capability/motivation barriers in February-March 2021 (n=1314), where participants chose their top 3 barriers to testing to view a tailored intervention. Phase 4 was a randomised experiment testing 2 audio-visual interventions addressing common testing barriers for people with lower health literacy in November 2021, targeting young adults as a key group endorsing misinformation (n=1527)."

1b-v) CONCLUSIONS/DISCUSSION in abstract for negative trials

Conclusions/Discussions in abstract for negative trials: Discuss the primary outcome - if the trial is negative (primary outcome not changed), and the intervention was not used, discuss whether negative results are attributable to lack of uptake and discuss reasons. (Note: Only report in the abstract what the main paper is reporting. If this information is missing from the main body of text, consider adding it)

	1	2	3	4	5	
subitem not at all important	○	○	◉	○	○	essential

Clear selection

Does your paper address subitem 1b-v?

Copy and paste relevant sections from the manuscript abstract (include quotes in quotation marks "like this" to indicate direct quotes from your manuscript), or elaborate on this item by providing additional information not in the ms, or briefly explain why the item is not applicable/relevant for your study

"In Phase 1, barriers were identified in all three categories of the COM-B model: capability (e.g. understanding which symptoms to test for), opportunity (e.g. not being able to access a PCR test), and motivation (e.g. not believing the symptoms could be COVID-19). Phase 2 found knowledge gaps for people with lower vs higher health literacy. Phase 3 found no differences between the intervention (health literacy-sensitive text using a wide range of barriers) and control groups. Phase 4 used a shorter intervention and showed that a fact-based animation, or a Tiktok video intervention presenting the same facts in a humorous style, increased knowledge about COVID-19 testing compared to government information. However, no differences were found for COVID-19 testing intentions."

INTRODUCTION

2a) In INTRODUCTION: Scientific background and explanation of rationale

2a-i) Problem and the type of system/solution

Describe the problem and the type of system/solution that is object of the study: intended as stand-alone intervention vs. incorporated in broader health care program? Intended for a particular patient population? Goals of the intervention, e.g., being more cost-effective to other interventions, replace or complement other solutions? (Note: Details about the intervention are provided in “Methods” under 5)

	1	2	3	4	5	
subitem not at all important	○	○	○	◉	○	essential

Clear selection

Does your paper address subitem 2a-i? *

Copy and paste relevant sections from the manuscript (include quotes in quotation marks "like this" to indicate direct quotes from your manuscript), or elaborate on this item by providing additional information not in the ms, or briefly explain why the item is not applicable/relevant for your study

"This program of work aimed to develop and test an intervention to address COVID-19 PCR testing barriers, to address the varying health literacy needs of the community. The intervention was developed and evaluated through 4 phases from June 2020 to November 2021.

- Phase 1, in June 2020, aimed to identify the range of barriers to COVID-19 testing.
- Phase 2, in November 2020, aimed to estimate the prevalence of barriers to COVID-19 testing, to target interventions to the most important issues.
- Phase 3, in February-March 2021, aimed to test the efficacy of providing health literacy-sensitive written information (i.e. adapted for people with lower health literacy) for all capability and motivation barriers identified in Phase 2, where individuals could view information to make a plan for their top 3 barriers to testing.
- Phase 4, in November 2021, aimed to address design issues in Phase 3 and test the efficacy of providing health literacy-sensitive audio-visual interventions (simple animation or Tiktok style video) for a smaller selection of common barriers for people with lower health literacy, identified in Phase 2."

2a-ii) Scientific background, rationale: What is known about the (type of) system

Scientific background, rationale: What is known about the (type of) system that is the object of the study (be sure to discuss the use of similar systems for other conditions/diagnoses, if appropiate), motivation for the study, i.e. what are the reasons for and what is the context for this specific study, from which stakeholder viewpoint is the study performed, potential impact of findings [2]. Briefly justify the choice of the comparator.

	1	2	3	4	5	
subitem not at all important	○	○	○	◉	○	essential

Clear selection

Does your paper address subitem 2a-ii? *

Copy and paste relevant sections from the manuscript (include quotes in quotation marks "like this" to indicate direct quotes from your manuscript), or elaborate on this item by providing additional information not in the ms, or briefly explain why the item is not applicable/relevant for your study

"The behaviour of individuals has been crucial to the control of COVID-19, from self isolating and testing to vaccination uptake[1]. A key prevention behaviour in early stages of the pandemic was polymerase chain reaction (PCR) testing for COVID-19[2]. In 2020-21, COVID-19 prevention strategies were often reliant on people getting a PCR test. This could be required when community members had been in contact with a positive case, had COVID-19 symptoms (e.g. fever, cough, sore throat), needed to travel from an outbreak area to another region, and for certain professions (e.g. health workers). In Australia, community members were required to self-isolate at home until they returned a negative PCR test result, and this test-trace-isolate strategy was used to determine the need for short-term localised restrictions until linked clusters of cases were brought under control[3].
In early 2020 there was little research on COVID-19 testing behaviours given the very new nature of this issue, but media reports suggested different barriers existed across countries, which was confirmed in subsequent research. "

2b) In INTRODUCTION: Specific objectives or hypotheses

Does your paper address CONSORT subitem 2b? *

Copy and paste relevant sections from the manuscript (include quotes in quotation marks "like this" to indicate direct quotes from your manuscript), or elaborate on this item by providing additional information not in the ms, or briefly explain why the item is not applicable/relevant for your study

"This program of work aimed to develop and test an intervention to address COVID-19 PCR testing barriers, to address the varying health literacy needs of the community. The intervention was developed and evaluated through 4 phases from June 2020 to November 2021.

- Phase 1, in June 2020, aimed to identify the range of barriers to COVID-19 testing.
- Phase 2, in November 2020, aimed to estimate the prevalence of barriers to COVID-19 testing, to target interventions to the most important issues.
- Phase 3, in February-March 2021, aimed to test the efficacy of providing health literacy-sensitive written information (i.e. adapted for people with lower health literacy) for all capability and motivation barriers identified in Phase 2, where individuals could view information to make a plan for their top 3 barriers to testing.
- Phase 4, in November 2021, aimed to address design issues in Phase 3 and test the efficacy of providing health literacy-sensitive audio-visual interventions (simple animation or Tiktok style video) for a smaller selection of common barriers for people with lower health literacy, identified in Phase 2."

METHODS

3a) Description of trial design (such as parallel, factorial) including allocation ratio

Does your paper address CONSORT subitem 3a? *

Copy and paste relevant sections from the manuscript (include quotes in quotation marks "like this" to indicate direct quotes from your manuscript), or elaborate on this item by providing additional information not in the ms, or briefly explain why the item is not applicable/relevant for your study

"Study design
Phase 1 and 2 both used a national survey study design. Phase 1 involved a survey of social media users using basic descriptive statistics. Phase 2 recruited a more representative sample via an online market research panel company with analysis comparing people with lower versus higher health literacy. Phase 3 and 4 used a randomised experiment design. Phase 3 used a 2 x 2 factorial design for COVID-19 testing intentions (2 groups compared post-intervention) and behavior (2 groups compared after 4 weeks), summarised in figure 1. Phase 4 used a 3 arm trial design for COVID-19 testing intentions (3 groups compared post-intervention) and behavior after 4 weeks, summarised in figure 1."

3b) Important changes to methods after trial commencement (such as eligibility criteria), with reasons

Does your paper address CONSORT subitem 3b? *

Copy and paste relevant sections from the manuscript (include quotes in quotation marks "like this" to indicate direct quotes from your manuscript), or elaborate on this item by providing additional information not in the ms, or briefly explain why the item is not applicable/relevant for your study

Not applicable

Your answer must have a minimum of 25 characters.

3b-i) Bug fixes, Downtimes, Content Changes

Bug fixes, Downtimes, Content Changes: ehealth systems are often dynamic systems. A description of changes to methods therefore also includes important changes made on the intervention or comparator during the trial (e.g., major bug fixes or changes in the functionality or content) (5-iii) and other “unexpected events” that may have influenced study design such as staff changes, system failures/downtimes, etc. [2].

	1	2	3	4	5	
subitem not at all important	○	○	◉	○	○	essential

Clear selection

Does your paper address subitem 3b-i?

Copy and paste relevant sections from the manuscript (include quotes in quotation marks "like this" to indicate direct quotes from your manuscript), or elaborate on this item by providing additional information not in the ms, or briefly explain why the item is not applicable/relevant for your study

Not applicable

Your answer must have a minimum of 25 characters.

4a) Eligibility criteria for participants

Does your paper address CONSORT subitem 4a? *

Copy and paste relevant sections from the manuscript (include quotes in quotation marks "like this" to indicate direct quotes from your manuscript), or elaborate on this item by providing additional information not in the ms, or briefly explain why the item is not applicable/relevant for your study

"This included any adults aged 18+, with quota sampling based on age, gender and education groups.
In Phase 4, social media users aged 18-39 on Facebook and Instagram were targeted with ads and further participants were recruited via the online panel company, with quota sampling based on education. Different states in Australia were targeted at different times of recruitment, so that participants were only recruited when there were very few or no cases in their state (when a test-trace-isolate strategy can be effective for containing spread)."

4a-i) Computer / Internet literacy

Computer / Internet literacy is often an implicit "de facto" eligibility criterion - this should be explicitly clarified.

	1	2	3	4	5	
subitem not at all important	○	○	◉	○	○	essential

Clear selection

Does your paper address subitem 4a-i?

Copy and paste relevant sections from the manuscript (include quotes in quotation marks "like this" to indicate direct quotes from your manuscript), or elaborate on this item by providing additional information not in the ms, or briefly explain why the item is not applicable/relevant for your study

Intervention open to anyone as a video to view; health literacy was measured

4a-ii) Open vs. closed, web-based vs. face-to-face assessments:

Open vs. closed, web-based vs. face-to-face assessments: Mention how participants were recruited (online vs. offline), e.g., from an open access website or from a clinic, and clarify if this was a purely web-based trial, or there were face-to-face components (as part of the intervention or for assessment), i.e., to what degree got the study team to know the participant. In online-only trials, clarify if participants were quasi-anonymous and whether having multiple identities was possible or whether technical or logistical measures (e.g., cookies, email confirmation, phone calls) were used to detect/prevent these.

	1	2	3	4	5	
subitem not at all important	○	○	○	○	◉	essential

Clear selection

Does your paper address subitem 4a-ii? *

Copy and paste relevant sections from the manuscript (include quotes in quotation marks "like this" to indicate direct quotes from your manuscript), or elaborate on this item by providing additional information not in the ms, or briefly explain why the item is not applicable/relevant for your study

"In Phase 1 social media users were asked to participate in a series of 10-minute surveys, with COVID-19 testing questions included in June 2020.
Phase 2 recruited a nationally representative sample, where eligible panel members were invited to participate through the company's usual channels.
A similar procedure was used for Phases 3 and 4 with recruitment through an online panel company
All data were collected and stored in anonymous format, but participants could provide contact details to receive compensation via points for online panel members and prize draws for gift vouchers if recruited via social media."

4a-iii) Information giving during recruitment

Information given during recruitment. Specify how participants were briefed for recruitment and in the informed consent procedures (e.g., publish the informed consent documentation as appendix, see also item X26), as this information may have an effect on user self-selection, user expectation and may also bias results.

	1	2	3	4	5	
subitem not at all important	○	○	○	○	◉	essential

Clear selection

Does your paper address subitem 4a-iii?

Copy and paste relevant sections from the manuscript (include quotes in quotation marks "like this" to indicate direct quotes from your manuscript), or elaborate on this item by providing additional information not in the ms, or briefly explain why the item is not applicable/relevant for your study

"After indicating their informed consent, participants completed a 10 minute online survey"

4b) Settings and locations where the data were collected

Does your paper address CONSORT subitem 4b? *

Copy and paste relevant sections from the manuscript (include quotes in quotation marks "like this" to indicate direct quotes from your manuscript), or elaborate on this item by providing additional information not in the ms, or briefly explain why the item is not applicable/relevant for your study

"In Phase 1 social media users were asked to participate in a series of 10-minute surveys, with COVID-19 testing questions included in June 2020.
Phase 2 recruited a nationally representative sample, where eligible panel members were invited to participate through the company's usual channels.
A similar procedure was used for Phases 3 and 4 with recruitment through an online panel company.
In Phase 4, social media users aged 18-39 on Facebook and Instagram were targeted with ads and further participants were recruited via the online panel company, with quota sampling based on education. Different states in Australia were targeted at different times of recruitment, so that participants were only recruited when there were very few or no cases in their state (when a test-trace-isolate strategy can be effective for containing spread)."

4b-i) Report if outcomes were (self-)assessed through online questionnaires

Clearly report if outcomes were (self-)assessed through online questionnaires (as common in web-based trials) or otherwise.

	1	2	3	4	5	
subitem not at all important	○	○	○	○	◉	essential

Clear selection

Does your paper address subitem 4b-i? *

Copy and paste relevant sections from the manuscript (include quotes in quotation marks "like this" to indicate direct quotes from your manuscript), or elaborate on this item by providing additional information not in the ms, or briefly explain why the item is not applicable/relevant for your study

"Survey questions for Phases 1 and 2 are provided in supplementary file 1. In each Phase we measured variables shown to be associated with differences in understanding of COVID-19 symptoms and prevention measures in our previous research [13], [18]: age, gender, language, health literacy, trust, living alone, prior COVID test. For the trials, our primary outcome was intention to undergo testing for COVID-19 if symptomatic (Table 3) (measured in a broad way for Phase 3, and a more specific way for Phase 4 to increase sensitivity). Secondary outcomes included intentions about other prevention behaviours (self-isolation if symptomatic, social distancing 1.5 meters, washing hands regularly, wearing masks in crowded indoor areas), understanding of messaging, risk perceptions, social stigma, and self-efficacy (i.e. confidence in overcoming perceived barriers to testing). In Phase 3 only, self-reported prevention behaviour and intentions were assessed after 1 month, with our prior survey data suggesting >20% of participants would experience symptoms over that time. See Table 1."

4b-ii) Report how institutional affiliations are displayed

Report how institutional affiliations are displayed to potential participants [on ehealth media], as affiliations with prestigious hospitals or universities may affect volunteer rates, use, and reactions with regards to an intervention.(Not a required item – describe only if this may bias results)

	1	2	3	4	5	
subitem not at all important	(●)	( )	( )	( )	( )	essential

Clear selection

Does your paper address subitem 4b-ii?

Copy and paste relevant sections from the manuscript (include quotes in quotation marks "like this" to indicate direct quotes from your manuscript), or elaborate on this item by providing additional information not in the ms, or briefly explain why the item is not applicable/relevant for your study

Not applicable

Your answer must have a minimum of 25 characters.

5) The interventions for each group with sufficient details to allow replication, including how and when they were actually administered

5-i) Mention names, credential, affiliations of the developers, sponsors, and owners

Mention names, credential, affiliations of the developers, sponsors, and owners [6] (if authors/evaluators are owners or developer of the software, this needs to be declared in a “Conflict of interest” section or mentioned elsewhere in the manuscript).

	1	2	3	4	5	
subitem not at all important	○	○	○	○	○	essential

Does your paper address subitem 5-i?

Copy and paste relevant sections from the manuscript (include quotes in quotation marks "like this" to indicate direct quotes from your manuscript), or elaborate on this item by providing additional information not in the ms, or briefly explain why the item is not applicable/relevant for your study

"The aim of Phase 4 was to develop a more targeted communication intervention with further refined testing outcome measures. We selected 4 capability (knowledge) barriers from the most prevalent issues for people with lower health literacy in Phase 2 and developed two audio-visual intervention scripts to address these: a simple animation in the style of Australian government advertisements, and a Tiktok-style video developed from the same information by a pharmacist with a large online following for COVID-19 information videos.
We thank the participants of the longitudinal COVID-19 survey for their ongoing participation in this research, Wendy Liang for consumer input for the written intervention, and Mustafa Dhahir for developing the Tiktok version of the intervention. The surveys were not specifically funded, but in-kind support was provided by authors with research fellowships. The experimental trials were funded by the Marie Bashir Institute (now named the Sydney Institute for Infectious Diseases) at the University of Sydney.
Carissa Bonner is supported by a National Health and Medical Research Council (NHMRC)/Heart Foundation Early Career Fellowship (#1122788).
Kirsten McCaffery is supported by a National Health and Medical Research Council (NHMRC) Principal Research Fellowship (#1121110)."

5-ii) Describe the history/development process

Describe the history/development process of the application and previous formative evaluations (e.g., focus groups, usability testing), as these will have an impact on adoption/use rates and help with interpreting results.

	1	2	3	4	5	
subitem not at all important	○	○	○	○	◉	essential

Clear selection

Does your paper address subitem 5-ii?

Copy and paste relevant sections from the manuscript (include quotes in quotation marks "like this" to indicate direct quotes from your manuscript), or elaborate on this item by providing additional information not in the ms, or briefly explain why the item is not applicable/relevant for your study

The entire paper addresses this through 4 phases of understanding and addressing the barriers to COVID testing

5-iii) Revisions and updating

Revisions and updating. Clearly mention the date and/or version number of the application/intervention (and comparator, if applicable) evaluated, or describe whether the intervention underwent major changes during the evaluation process, or whether the development and/or content was "frozen" during the trial. Describe dynamic components such as news feeds or changing content which may have an impact on the replicability of the intervention (for unexpected events see item 3b).

	1	2	3	4	5	
subitem not at all important	○	○	◉	○	○	essential

Clear selection

Does your paper address subitem 5-iii?

Copy and paste relevant sections from the manuscript (include quotes in quotation marks "like this" to indicate direct quotes from your manuscript), or elaborate on this item by providing additional information not in the ms, or briefly explain why the item is not applicable/relevant for your study

Not applicable; content is unchanged and provided in screenshots/transcripts/URL links

5-iv) Quality assurance methods

Provide information on quality assurance methods to ensure accuracy and quality of information provided [1], if applicable.

	1	2	3	4	5	
subitem not at all important	○	○	●	○	○	essential

Clear selection

Does your paper address subitem 5-iv?

Copy and paste relevant sections from the manuscript (include quotes in quotation marks "like this" to indicate direct quotes from your manuscript), or elaborate on this item by providing additional information not in the ms, or briefly explain why the item is not applicable/relevant for your study

Anonymous data is available from the authors.

5-v) Ensure replicability by publishing the source code, and/or providing screenshots/screen-capture video, and/or providing flowcharts of the algorithms used

Ensure replicability by publishing the source code, and/or providing screenshots/screen-capture video, and/or providing flowcharts of the algorithms used. Replicability (i.e., other researchers should in principle be able to replicate the study) is a hallmark of scientific reporting.

	1	2	3	4	5	
subitem not at all important	○	○	○	○	●	essential

Clear selection

Does your paper address subitem 5-v?

Copy and paste relevant sections from the manuscript (include quotes in quotation marks "like this" to indicate direct quotes from your manuscript), or elaborate on this item by providing additional information not in the ms, or briefly explain why the item is not applicable/relevant for your study

Multimedia appendix provides URLs, transcripts and screenshots
"Images and text for the Phase 4 intervention are provided in supplementary file 3. There were two audio-visual interventions: one was an animation and the other was a TikTok-style video, which was more humorous. Both covered the following barriers to COVID-19 testing, which were identified as the most prevalent knowledge issues for people with lower health literacy in Phase 2:

- I know what symptoms I have and don't believe they are COVID-19 ones e.g. hayfever/normal cold
- I'm not sure my symptoms are bad enough
- It is unlikely I have COVID-19 because there aren't many cases in my area
- I'm not sure this symptom needs testing"

5-vi) Digital preservation

Digital preservation: Provide the URL of the application, but as the intervention is likely to change or disappear over the course of the years; also make sure the intervention is archived (Internet Archive, webcitation.org, and/or publishing the source code or screenshots/videos alongside the article). As pages behind login screens cannot be archived, consider creating demo pages which are accessible without login.

	1	2	3	4	5	
subitem not at all important	○	○	○	●	○	essential

Clear selection

Does your paper address subitem 5-vi?

Copy and paste relevant sections from the manuscript (include quotes in quotation marks "like this" to indicate direct quotes from your manuscript), or elaborate on this item by providing additional information not in the ms, or briefly explain why the item is not applicable/relevant for your study

Multimedia appendix provides URLs, transcripts and screenshots
"Images and text for the Phase 4 intervention are provided in supplementary file 3. There were two audio-visual interventions: one was an animation and the other was a TikTok-style video, which was more humorous. Both covered the following barriers to COVID-19 testing, which were identified as the most prevalent knowledge issues for people with lower health literacy in Phase 2:
• I know what symptoms I have and don't believe they are COVID-19 ones e.g. hayfever/normal cold
• I'm not sure my symptoms are bad enough
• It is unlikely I have COVID-19 because there aren't many cases in my area
• I'm not sure this symptom needs testing"

5-vii) Access

Access: Describe how participants accessed the application, in what setting/context, if they had to pay (or were paid) or not, whether they had to be a member of specific group. If known, describe how participants obtained "access to the platform and Internet" [1]. To ensure access for editors/reviewers/readers, consider to provide a "backdoor" login account or demo mode for reviewers/readers to explore the application (also important for archiving purposes, see vi).

	1	2	3	4	5	
subitem not at all important	○	○	◉	○	○	essential

Clear selection

Does your paper address subitem 5-vii? *

Copy and paste relevant sections from the manuscript (include quotes in quotation marks "like this" to indicate direct quotes from your manuscript), or elaborate on this item by providing additional information not in the ms, or briefly explain why the item is not applicable/relevant for your study

"Phase 1 and 2 both used a national survey study design. Phase 1 involved a survey of social media users using basic descriptive statistics. Phase 2 recruited a more representative sample via an online market research panel company with analysis comparing people with lower versus higher health literacy. Phase 3 and 4 used a randomised experiment design. Phase 3 used a 2 x 2 factorial design for COVID-19 testing intentions (2 groups compared post-intervention) and behavior (2 groups compared after 4 weeks), summarised in figure 1. Phase 4 used a 3 arm trial design for COVID-19 testing intentions (3 groups compared post-intervention) and behavior after 4 weeks, summarised in figure 1."

5-viii) Mode of delivery, features/functionalities/components of the intervention and comparator, and the theoretical framework

Describe mode of delivery, features/functionalities/components of the intervention and comparator, and the theoretical framework [6] used to design them (instructional strategy [1], behaviour change techniques, persuasive features, etc., see e.g., [7, 8] for terminology). This includes an in-depth description of the content (including where it is coming from and who developed it) [1]," whether [and how] it is tailored to individual circumstances and allows users to track their progress and receive feedback" [6]. This also includes a description of communication delivery channels and – if computer-mediated communication is a component – whether communication was synchronous or asynchronous [6]. It also includes information on presentation strategies [1], including page design principles, average amount of text on pages, presence of hyperlinks to other resources, etc. [1].

	1	2	3	4	5	
subitem not at all important	○	○	○	◉	○	essential

Clear selection

Does your paper address subitem 5-viii? *

Copy and paste relevant sections from the manuscript (include quotes in quotation marks "like this" to indicate direct quotes from your manuscript), or elaborate on this item by providing additional information not in the ms, or briefly explain why the item is not applicable/relevant for your study

"Theoretical framework: According to the Capability-Opportunity-Motivation-Behaviour (COM-B) model[15], health prevention behaviours can be conceptualised in terms of three main drivers: physical/psychological capability (e.g. having the physical ability to drive to or walk up the stairs to access a testing centre, and knowing what to do if you have symptoms), physical/social opportunity (e.g. the availability of testing centres in your area, and social norms that make testing and self-isolation acceptable), and automatic/reflective motivation (e.g. fear of a painful test, an explicit belief that it's important to get tested for symptoms)[2]. In early 2020 we used this framework as the basis for a new program of research on the novel behaviour of COVID-19 PCR testing."

"Images and text for the intervention in Phase 3 are provided in supplementary file 2. The health literacy-sensitive version of the text was developed by applying health literacy guidelines [19], using an online tool that provides objective feedback on the complexity of health information (e.g. grade reading score, passive voice, medical jargon)[20], and by incorporating consumer feedback. We used the Sydney Health Literacy Lab (SHeLL) Editor developed by our team [20] to meet the recommended Grade 8 school level, by simplifying complex words, sentences and grammar. Participants randomised to the intervention were asked to choose their top 3 barriers to testing, then selected 1 barrier for the health literacy-sensitive action plan. This was adapted from our previous studies on health-related lifestyle change to address intention-behaviour gaps[21], [22]. The online action plan used an 'if-then' format (e.g. 'If I don't want to get tested because there aren't many cases in my area, then I will remind myself that every new outbreak of COVID-19 starts with one new case'), a format which has shown to improve various behavioural outcomes including smoking cessation, physical activity, and healthy eating[23]–[30]. Health literacy principles were applied to the if-then plan (e.g. simple language, images, breaking down tasks into smaller steps) as previous research has shown that this can improve the effectiveness of if-then plans for people with low health literacy[21], [31]. The 'if' options reflect the top 10 barriers for people with lower health literacy identified in Phase 2:

- I would prefer to isolate instead
- I'm not sure this symptom is one that needs testing
- I have symptoms of COVID-19 but I don't think they are bad enough
- I have symptoms of COVID-19 but I think it's a cold or hay fever
- I'm worried the test is painful
- I'm worried about spreading my illness on the way to the testing centre
- There aren't many cases in my area
- I'm worried I will catch COVID-19 when I get tested or on the way to the testing centre
- I'm not sure what to do
- I'd like my doctor's advice first

Participants could then select a solution ('then' option). Solutions were generated in collaboration with our consumer representative.

Images and text for the Phase 4 intervention are provided in supplementary file 3. There were two audio-visual interventions: one was an animation and the other was a TikTok-style video, which was more humorous. Both covered the following barriers to COVID-19 testing, which were identified as the most prevalent knowledge issues for people with lower health literacy in Phase 2:

- I know what symptoms I have and don't believe they are COVID-19 ones e.g.

hayfever/normal cold
- I'm not sure my symptoms are bad enough
- It is unlikely I have COVID-19 because there aren't many cases in my area
- I'm not sure this symptom needs testing"

5-ix) Describe use parameters

Describe use parameters (e.g., intended “doses” and optimal timing for use). Clarify what instructions or recommendations were given to the user, e.g., regarding timing, frequency, heaviness of use, if any, or was the intervention used ad libitum.

	1	2	3	4	5	
subitem not at all important	○	○	◉	○	○	essential

Clear selection

Does your paper address subitem 5-ix?

Copy and paste relevant sections from the manuscript (include quotes in quotation marks "like this" to indicate direct quotes from your manuscript), or elaborate on this item by providing additional information not in the ms, or briefly explain why the item is not applicable/relevant for your study

"Those randomised to the intervention groups viewed a 2 minute audio-visual intervention, while those in the control group viewed standard written government information. Access to the outcome questions to complete the survey was enabled after 73 seconds for the animation and 65 seconds for the TikTok (the lengths of the audio-visual intervention) to increase the chance that participants viewed the intervention."

5-x) Clarify the level of human involvement

Clarify the level of human involvement (care providers or health professionals, also technical assistance) in the e-intervention or as co-intervention (detail number and expertise of professionals involved, if any, as well as “type of assistance offered, the timing and frequency of the support, how it is initiated, and the medium by which the assistance is delivered”. It may be necessary to distinguish between the level of human involvement required for the trial, and the level of human involvement required for a routine application outside of a RCT setting (discuss under item 21 – generalizability).

	1	2	3	4	5	
subitem not at all important	○	○	◉	○	○	essential

Clear selection

Does your paper address subitem 5-x?

Copy and paste relevant sections from the manuscript (include quotes in quotation marks "like this" to indicate direct quotes from your manuscript), or elaborate on this item by providing additional information not in the ms, or briefly explain why the item is not applicable/relevant for your study

Not applicable - videos only

5-xi) Report any prompts/reminders used

Report any prompts/reminders used: Clarify if there were prompts (letters, emails, phone calls, SMS) to use the application, what triggered them, frequency etc. It may be necessary to distinguish between the level of prompts/reminders required for the trial, and the level of prompts/reminders for a routine application outside of a RCT setting (discuss under item 21 – generalizability).

	1	2	3	4	5	
subitem not at all important	○	○	○	◉	○	essential

Clear selection

Does your paper address subitem 5-xi? *

Copy and paste relevant sections from the manuscript (include quotes in quotation marks "like this" to indicate direct quotes from your manuscript), or elaborate on this item by providing additional information not in the ms, or briefly explain why the item is not applicable/relevant for your study

"In Phase 3, they were randomised to view written government information or the intervention, and completed outcome questions. Those randomised to the intervention selected three relevant barriers and viewed health literacy-sensitive information about those issues, and created a plan for one chosen barrier. They were asked to create an action plan to help them overcome their barriers, and received weekly reminders with a screenshot of their action plan by email. After 4 weeks, all participants received a 5 minute online survey (see Table 3).

In Phase 4, social media users aged 18-39 on Facebook and Instagram were targeted with ads and further participants were recruited via the online panel company, with quota sampling based on education. Different states in Australia were targeted at different times of recruitment, so that participants were only recruited when there were very few or no cases in their state (when a test-trace-isolate strategy can be effective for containing spread). The following advertisement text was used for social media: "We want to hear from you! Complete a short survey about COVID-19 and be in with the chance to win a $20 gift card." All participants answered a 10 minute survey (see Table 4). Those randomised to the intervention groups viewed a 2 minute audio-visual intervention, while those in the control group viewed standard written government information. Access to the outcome questions to complete the survey was enabled after 73 seconds for the animation and 65 seconds for the TikTok (the lengths of the audio-visual intervention) to increase the chance that participants viewed the intervention."

5-xii) Describe any co-interventions (incl. training/support)

Describe any co-interventions (incl. training/support): Clearly state any interventions that are provided in addition to the targeted eHealth intervention, as ehealth intervention may not be designed as stand-alone intervention. This includes training sessions and support [1]. It may be necessary to distinguish between the level of training required for the trial, and the level of training for a routine application outside of a RCT setting (discuss under item 21 – generalizability.

	1	2	3	4	5	
subitem not at all important	○	○	◉	○	○	essential

Clear selection

Does your paper address subitem 5-xii? *

Copy and paste relevant sections from the manuscript (include quotes in quotation marks "like this" to indicate direct quotes from your manuscript), or elaborate on this item by providing additional information not in the ms, or briefly explain why the item is not applicable/relevant for your study

Not applicable - online interventions within surveys only

6a) Completely defined pre-specified primary and secondary outcome measures, including how and when they were assessed

Does your paper address CONSORT subitem 6a? *

Copy and paste relevant sections from the manuscript (include quotes in quotation marks "like this" to indicate direct quotes from your manuscript), or elaborate on this item by providing additional information not in the ms, or briefly explain why the item is not applicable/relevant for your study

"Outcomes
Survey questions for Phases 1 and 2 are provided in supplementary file 1. In each Phase we measured variables shown to be associated with differences in understanding of COVID-19 symptoms and prevention measures in our previous research [13], [18]: age, gender, language, health literacy, trust, living alone, prior COVID test. For the trials, our primary outcome was intention to undergo testing for COVID-19 if symptomatic (Table 3) (measured in a broad way for Phase 3, and a more specific way for Phase 4 to increase sensitivity). Secondary outcomes included intentions about other prevention behaviours (self-isolation if symptomatic, social distancing 1.5 meters, washing hands regularly, wearing masks in crowded indoor areas), understanding of messaging, risk perceptions, social stigma, and self-efficacy (i.e. confidence in overcoming perceived barriers to testing). In Phase 3 only, self-reported prevention behaviour and intentions were assessed after 1 month, with our prior survey data suggesting >20% of participants would experience symptoms over that time. See Table 1."

6a-i) Online questionnaires: describe if they were validated for online use and apply CHERRIES items to describe how the questionnaires were designed/deployed

If outcomes were obtained through online questionnaires, describe if they were validated for online use and apply CHERRIES items to describe how the questionnaires were designed/deployed [9].

	1	2	3	4	5	
subitem not at all important	○	○	◉	○	○	essential

Clear selection

Does your paper address subitem 6a-i?

Copy and paste relevant sections from manuscript text

Validated outcomes are indicated in table 1 as described above.

6a-ii) Describe whether and how "use" (including intensity of use/dosage) was defined/measured/monitored

Describe whether and how "use" (including intensity of use/dosage) was defined/measured/monitored (logins, logfile analysis, etc.). Use/adoption metrics are important process outcomes that should be reported in any ehealth trial.

	1	2	3	4	5	
subitem not at all important	○	○	◉	○	○	essential

Clear selection

Does your paper address subitem 6a-ii?

Copy and paste relevant sections from manuscript text

One off interventions; further use/access not evaluated

6a-iii) Describe whether, how, and when qualitative feedback from participants was obtained

Describe whether, how, and when qualitative feedback from participants was obtained (e.g., through emails, feedback forms, interviews, focus groups).

	1	2	3	4	5	
subitem not at all important	○	○	◉	○	○	essential

Clear selection

Does your paper address subitem 6a-iii?

Copy and paste relevant sections from manuscript text

"Table 2 maps all the barriers identified in open survey responses to the COM-B drivers of behaviour (capability, opportunity and motivation)."

6b) Any changes to trial outcomes after the trial commenced, with reasons

Does your paper address CONSORT subitem 6b? *

Copy and paste relevant sections from the manuscript (include quotes in quotation marks "like this" to indicate direct quotes from your manuscript), or elaborate on this item by providing additional information not in the ms, or briefly explain why the item is not applicable/relevant for your study

Not applicable, trial was pre-registered

7a) How sample size was determined

NPT: When applicable, details of whether and how the clustering by care provides or centers was addressed

7a-i) Describe whether and how expected attrition was taken into account when calculating the sample size

Describe whether and how expected attrition was taken into account when calculating the sample size.

	1	2	3	4	5	
subitem not at all important	○	○	●	○	○	essential

Clear selection

Does your paper address subitem 7a-i?

Copy and paste relevant sections from manuscript title (include quotes in quotation marks "like this" to indicate direct quotes from your manuscript), or elaborate on this item by providing additional information not in the ms, or briefly explain why the item is not applicable/relevant for your study

"the experiments were pre-registered on the Australia New Zealand Trial Registry (ACTRN12621000876897[16]; ACTRN12620001355965[17]).
After 4 weeks, 60% of respondents completed follow-up measures (n=790)"

7b) When applicable, explanation of any interim analyses and stopping guidelines

Does your paper address CONSORT subitem 7b? *

Copy and paste relevant sections from the manuscript (include quotes in quotation marks "like this" to indicate direct quotes from your manuscript), or elaborate on this item by providing additional information not in the ms, or briefly explain why the item is not applicable/relevant for your study

Not applicable - one analysis point only

8a) Method used to generate the random allocation sequence

NPT: When applicable, how care providers were allocated to each trial group

Does your paper address CONSORT subitem 8a? *

Copy and paste relevant sections from the manuscript (include quotes in quotation marks "like this" to indicate direct quotes from your manuscript), or elaborate on this item by providing additional information not in the ms, or briefly explain why the item is not applicable/relevant for your study

"A similar procedure was used for Phases 3 and 4 with recruitment through an online panel company, but participants were randomised to view different versions of COVID-19 testing information. In Phase 3, they were randomised to view written government information or the intervention, and completed outcome questions. Those randomised to the intervention selected three relevant barriers and viewed health literacy-sensitive information about those issues, and created a plan for one chosen barrier. They were asked to create an action plan to help them overcome their barriers, and received weekly reminders with a screenshot of their action plan by email. After 4 weeks, all participants received a 5 minute online survey (see Table 3).

In Phase 4, social media users aged 18-39 on Facebook and Instagram were targeted with ads and further participants were recruited via the online panel company, with quota sampling based on education. Different states in Australia were targeted at different times of recruitment, so that participants were only recruited when there were very few or no cases in their state (when a test-trace-isolate strategy can be effective for containing spread). The following advertisement text was used for social media: "We want to hear from you! Complete a short survey about COVID-19 and be in with the chance to win a $20 gift card." All participants answered a 10 minute survey (see Table 4). Those randomised to the intervention groups viewed a 2 minute audio-visual intervention, while those in the control group viewed standard written government information. "

8b) Type of randomisation; details of any restriction (such as blocking and block size)

Does your paper address CONSORT subitem 8b? *

Copy and paste relevant sections from the manuscript (include quotes in quotation marks "like this" to indicate direct quotes from your manuscript), or elaborate on this item by providing additional information not in the ms, or briefly explain why the item is not applicable/relevant for your study

Not applicable - no restriction; equal groups

9) Mechanism used to implement the random allocation sequence (such as sequentially numbered containers), describing any steps taken to conceal the sequence until interventions were assigned

Does your paper address CONSORT subitem 9? *

Copy and paste relevant sections from the manuscript (include quotes in quotation marks "like this" to indicate direct quotes from your manuscript), or elaborate on this item by providing additional information not in the ms, or briefly explain why the item is not applicable/relevant for your study

Not applicable - randomization done within survey itself

10) Who generated the random allocation sequence, who enrolled participants, and who assigned participants to interventions

Does your paper address CONSORT subitem 10? *

Copy and paste relevant sections from the manuscript (include quotes in quotation marks "like this" to indicate direct quotes from your manuscript), or elaborate on this item by providing additional information not in the ms, or briefly explain why the item is not applicable/relevant for your study

Not applicable - randomization done within survey itself

11a) If done, who was blinded after assignment to interventions (for example, participants, care providers, those assessing outcomes) and how

NPT: Whether or not administering co-interventions were blinded to group assignment

11a-i) Specify who was blinded, and who wasn't

Specify who was blinded, and who wasn't. Usually, in web-based trials it is not possible to blind the participants [1, 3] (this should be clearly acknowledged), but it may be possible to blind outcome assessors, those doing data analysis or those administering co-interventions (if any).

	1	2	3	4	5	
subitem not at all important	○	◉	○	○	○	essential

Clear selection

Does your paper address subitem 11a-i? *

Copy and paste relevant sections from the manuscript (include quotes in quotation marks "like this" to indicate direct quotes from your manuscript), or elaborate on this item by providing additional information not in the ms, or briefly explain why the item is not applicable/relevant for your study

Not applicable - Blinding not possible for interactive interventions online

11a-ii) Discuss e.g., whether participants knew which intervention was the "intervention of interest" and which one was the "comparator"

Informed consent procedures (4a-ii) can create biases and certain expectations - discuss e.g., whether participants knew which intervention was the "intervention of interest" and which one was the "comparator".

	1	2	3	4	5	
subitem not at all important	○	○	◉	○	○	essential

Clear selection

Does your paper address subitem 11a-ii?

Copy and paste relevant sections from the manuscript (include quotes in quotation marks "like this" to indicate direct quotes from your manuscript), or elaborate on this item by providing additional information not in the ms, or briefly explain why the item is not applicable/relevant for your study

Not applicable - Blinding not possible for interactive interventions online

11b) If relevant, description of the similarity of interventions

(this item is usually not relevant for ehealth trials as it refers to similarity of a placebo or sham intervention to a active medication/intervention)

Does your paper address CONSORT subitem 11b? *

Copy and paste relevant sections from the manuscript (include quotes in quotation marks "like this" to indicate direct quotes from your manuscript), or elaborate on this item by providing additional information not in the ms, or briefly explain why the item is not applicable/relevant for your study

Not applicable as defined above - not medical

12a) Statistical methods used to compare groups for primary and secondary outcomes

NPT: When applicable, details of whether and how the clustering by care providers or centers was addressed

Does your paper address CONSORT subitem 12a? *

Copy and paste relevant sections from the manuscript (include quotes in quotation marks "like this" to indicate direct quotes from your manuscript), or elaborate on this item by providing additional information not in the ms, or briefly explain why the item is not applicable/relevant for your study

"Analyses
For Phases 1 and 2, participant characteristics and survey question responses are reported descriptively, using a content analysis approach for open responses and χ^2 tests to compare responses across health literacy levels. Two researchers mapped the text from open responses to components of the COM-B model, with discussion to resolve discrepancies. For Phases 3 and 4, analyses were conducted using planned contrasts between the intervention arms and control arm, implemented in regression models. Continuous outcomes were analysed using linear regression to estimate marginal mean differences; dichotomous outcomes were analysed using generalized linear models with a modified Poisson approach (log link, robust standard errors) to estimate relative risks; count variables were analysed using Poisson regression to estimate relative risks. In Phase 3, analyses controlled for age, gender, language, health literacy, trust, living alone, and previous COVID-19 testing. In Phase 4, positive baseline intention, age, gender, language, health literacy, trust, and perceived COVID-19 risk in Australia were controlled for. Interactions between health literacy and randomised condition were also explored."

12a-i) Imputation techniques to deal with attrition / missing values

Imputation techniques to deal with attrition / missing values: Not all participants will use the intervention/comparator as intended and attrition is typically high in ehealth trials. Specify how participants who did not use the application or dropped out from the trial were treated in the statistical analysis (a complete case analysis is strongly discouraged, and simple imputation techniques such as LOCF may also be problematic [4]).

	1	2	3	4	5	
subitem not at all important	○	●	○	○	○	essential

Clear selection

Does your paper address subitem 12a-i? *

Copy and paste relevant sections from the manuscript (include quotes in quotation marks "like this" to indicate direct quotes from your manuscript), or elaborate on this item by providing additional information not in the ms, or briefly explain why the item is not applicable/relevant for your study

Not applicable - no imputation

12b) Methods for additional analyses, such as subgroup analyses and adjusted analyses

Does your paper address CONSORT subitem 12b? *

Copy and paste relevant sections from the manuscript (include quotes in quotation marks "like this" to indicate direct quotes from your manuscript), or elaborate on this item by providing additional information not in the ms, or briefly explain why the item is not applicable/relevant for your study

"Interactions between health literacy and randomised condition were also explored."

X26) REB/IRB Approval and Ethical Considerations [recommended as subheading under "Methods"] (not a CONSORT item)

X26-i) Comment on ethics committee approval

	1	2	3	4	5	
subitem not at all important	○	○	○	○	●	essential

Clear selection

Does your paper address subitem X26-i?

Copy and paste relevant sections from the manuscript (include quotes in quotation marks "like this" to indicate direct quotes from your manuscript), or elaborate on this item by providing additional information not in the ms, or briefly explain why the item is not applicable/relevant for your study

"Ethics approval was obtained from the University of Sydney Human Research Ethics Committee (project number 2020/781), and the experiments were pre-registered on the Australia New Zealand Trial Registry (ACTRN12621000876897[16]; ACTRN12620001355965[17])."

x26-ii) Outline informed consent procedures

Outline informed consent procedures e.g., if consent was obtained offline or online (how? Checkbox, etc.?), and what information was provided (see 4a-ii). See [6] for some items to be included in informed consent documents.

	1	2	3	4	5	
subitem not at all important	○	○	○	●	○	essential

Clear selection

Does your paper address subitem X26-ii?

Copy and paste relevant sections from the manuscript (include quotes in quotation marks "like this" to indicate direct quotes from your manuscript), or elaborate on this item by providing additional information not in the ms, or briefly explain why the item is not applicable/relevant for your study

"All data were collected and stored in anonymous format,...After indicating their informed consent, participants completed a 10 minute online survey"

X26-iii) Safety and security procedures

Safety and security procedures, incl. privacy considerations, and any steps taken to reduce the likelihood or detection of harm (e.g., education and training, availability of a hotline)

	1	2	3	4	5	
subitem not at all important	○	○	◉	○	○	essential

Clear selection

Does your paper address subitem X26-iii?

Copy and paste relevant sections from the manuscript (include quotes in quotation marks "like this" to indicate direct quotes from your manuscript), or elaborate on this item by providing additional information not in the ms, or briefly explain why the item is not applicable/relevant for your study

"All data were collected and stored in anonymous format"

RESULTS

13a) For each group, the numbers of participants who were randomly assigned, received intended treatment, and were analysed for the primary outcome

NPT: The number of care providers or centers performing the intervention in each group and the number of patients treated by each care provider in each center

Does your paper address CONSORT subitem 13a? *

Copy and paste relevant sections from the manuscript (include quotes in quotation marks "like this" to indicate direct quotes from your manuscript), or elaborate on this item by providing additional information not in the ms, or briefly explain why the item is not applicable/relevant for your study

"Phase 1: Identification of COVID-19 testing barriers (June 2020, n=1369 Australian adults 18-90 years)
Phase 2: Prevalence of barriers to testing for COVID-19 (November 2020, n=2034 nationally representative sample of Australian adults 18-90 years)
Phase 3: Randomised experiment to test the effect of health literacy-sensitive written information about COVID-19 testing barriers (Feb-March 2021, n=1314). After 4 weeks, 60% of respondents completed follow-up measures (n=790),
Phase 4: Randomised experiment to test the effect of health literacy-sensitive audio-visual interventions about COVID-19 testing barriers in adults with lower health literacy (November 2021, n=1527 Australian adults aged 18-49 years)"

13b) For each group, losses and exclusions after randomisation, together with reasons

Does your paper address CONSORT subitem 13b? (NOTE: Preferably, this is shown in a CONSORT flow diagram) *

Copy and paste relevant sections from the manuscript (include quotes in quotation marks "like this" to indicate direct quotes from your manuscript), or elaborate on this item by providing additional information not in the ms, or briefly explain why the item is not applicable/relevant for your study

Participant characteristics provided in appendix

13b-i) Attrition diagram

Strongly recommended: An attrition diagram (e.g., proportion of participants still logging in or using the intervention/comparator in each group plotted over time, similar to a survival curve) or other figures or tables demonstrating usage/dose/engagement.

	1	2	3	4	5	
subitem not at all important	○	○	◉	○	○	essential

Clear selection

Does your paper address subitem 13b-i?

Copy and paste relevant sections from the manuscript or cite the figure number if applicable (include quotes in quotation marks "like this" to indicate direct quotes from your manuscript), or elaborate on this item by providing additional information not in the ms, or briefly explain why the item is not applicable/relevant for your study

Not applicable - one off intervention

14a) Dates defining the periods of recruitment and follow-up

Does your paper address CONSORT subitem 14a? *

Copy and paste relevant sections from the manuscript (include quotes in quotation marks "like this" to indicate direct quotes from your manuscript), or elaborate on this item by providing additional information not in the ms, or briefly explain why the item is not applicable/relevant for your study

"Phase 1: Identification of COVID-19 testing barriers (June 2020, n=1369 Australian adults 18-90 years)
Phase 2: Prevalence of barriers to testing for COVID-19 (November 2020, n=2034 nationally representative sample of Australian adults 18-90 years)
Phase 3: Randomised experiment to test the effect of health literacy-sensitive written information about COVID-19 testing barriers (Feb-March 2021, n=1314). After 4 weeks, 60% of respondents completed follow-up measures (n=790),
Phase 4: Randomised experiment to test the effect of health literacy-sensitive audio-visual interventions about COVID-19 testing barriers in adults with lower health literacy (November 2021, n=1527 Australian adults aged 18-49 years)"

14a-i) Indicate if critical “secular events” fell into the study period

Indicate if critical “secular events” fell into the study period, e.g., significant changes in Internet resources available or “changes in computer hardware or Internet delivery resources”

	1	2	3	4	5	
subitem not at all important	○	○	◉	○	○	essential

Clear selection

Does your paper address subitem 14a-i?

Copy and paste relevant sections from the manuscript (include quotes in quotation marks "like this" to indicate direct quotes from your manuscript), or elaborate on this item by providing additional information not in the ms, or briefly explain why the item is not applicable/relevant for your study

Not applicable - no changes

14b) Why the trial ended or was stopped (early)

Does your paper address CONSORT subitem 14b? *

Copy and paste relevant sections from the manuscript (include quotes in quotation marks "like this" to indicate direct quotes from your manuscript), or elaborate on this item by providing additional information not in the ms, or briefly explain why the item is not applicable/relevant for your study

Not applicable - not stopped

15) A table showing baseline demographic and clinical characteristics for each group

NPT: When applicable, a description of care providers (case volume, qualification, expertise, etc.) and centers (volume) in each group

Does your paper address CONSORT subitem 15? *

Copy and paste relevant sections from the manuscript (include quotes in quotation marks "like this" to indicate direct quotes from your manuscript), or elaborate on this item by providing additional information not in the ms, or briefly explain why the item is not applicable/relevant for your study

Appendix reports full sample characteristics for each phase

15-i) Report demographics associated with digital divide issues

In ehealth trials it is particularly important to report demographics associated with digital divide issues, such as age, education, gender, social-economic status, computer/Internet /ehealth literacy of the participants, if known.

	1	2	3	4	5	
subitem not at all important	○	○	○	◉	○	essential

Clear selection

Does your paper address subitem 15-i? *

Copy and paste relevant sections from the manuscript (include quotes in quotation marks "like this" to indicate direct quotes from your manuscript), or elaborate on this item by providing additional information not in the ms, or briefly explain why the item is not applicable/relevant for your study

Appendix reports full sample characteristics for each phase including health literacy and education

16) For each group, number of participants (denominator) included in each analysis and whether the analysis was by original assigned groups

16-i) Report multiple "denominators" and provide definitions

Report multiple "denominators" and provide definitions: Report N's (and effect sizes) "across a range of study participation [and use] thresholds" [1], e.g., N exposed, N consented, N used more than x times, N used more than y weeks, N participants "used" the intervention/comparator at specific pre-defined time points of interest (in absolute and relative numbers per group). Always clearly define "use" of the intervention.

	1	2	3	4	5	
subitem not at all important	○	○	◉	○	○	essential

Clear selection

Does your paper address subitem 16-i? *

Copy and paste relevant sections from the manuscript (include quotes in quotation marks "like this" to indicate direct quotes from your manuscript), or elaborate on this item by providing additional information not in the ms, or briefly explain why the item is not applicable/relevant for your study

Not applicable - one off intervention; but denominator for each phase is clearly defined throughout results tables

16-ii) Primary analysis should be intent-to-treat

Primary analysis should be intent-to-treat, secondary analyses could include comparing only "users", with the appropriate caveats that this is no longer a randomized sample (see 18-i).

	1	2	3	4	5	
subitem not at all important	○	○	○	◉	○	essential

Clear selection

Does your paper address subitem 16-ii?

Copy and paste relevant sections from the manuscript (include quotes in quotation marks "like this" to indicate direct quotes from your manuscript), or elaborate on this item by providing additional information not in the ms, or briefly explain why the item is not applicable/relevant for your study

See pre-registration details "the experiments were pre-registered on the Australia New Zealand Trial Registry (ACTRN12621000876897[16]; ACTRN12620001355965[17])."

17a) For each primary and secondary outcome, results for each group, and the estimated effect size and its precision (such as 95% confidence interval)

Does your paper address CONSORT subitem 17a? *

Copy and paste relevant sections from the manuscript (include quotes in quotation marks "like this" to indicate direct quotes from your manuscript), or elaborate on this item by providing additional information not in the ms, or briefly explain why the item is not applicable/relevant for your study

See results tables and appendices

17a-i) Presentation of process outcomes such as metrics of use and intensity of use

In addition to primary/secondary (clinical) outcomes, the presentation of process outcomes such as metrics of use and intensity of use (dose, exposure) and their operational definitions is critical. This does not only refer to metrics of attrition (13-b) (often a binary variable), but also to more continuous exposure metrics such as “average session length”. These must be accompanied by a technical description how a metric like a “session” is defined (e.g., timeout after idle time) [1] (report under item 6a).

	1	2	3	4	5	
subitem not at all important	○	○	●	○	○	essential

Clear selection

Does your paper address subitem 17a-i?

Copy and paste relevant sections from the manuscript (include quotes in quotation marks "like this" to indicate direct quotes from your manuscript), or elaborate on this item by providing additional information not in the ms, or briefly explain why the item is not applicable/relevant for your study

Not applicable - one off intervention

17b) For binary outcomes, presentation of both absolute and relative effect sizes is recommended

Does your paper address CONSORT subitem 17b? *

Copy and paste relevant sections from the manuscript (include quotes in quotation marks "like this" to indicate direct quotes from your manuscript), or elaborate on this item by providing additional information not in the ms, or briefly explain why the item is not applicable/relevant for your study

Not applicable - primary outcomes are continuous

18) Results of any other analyses performed, including subgroup analyses and adjusted analyses, distinguishing pre-specified from exploratory

Does your paper address CONSORT subitem 18? *

Copy and paste relevant sections from the manuscript (include quotes in quotation marks "like this" to indicate direct quotes from your manuscript), or elaborate on this item by providing additional information not in the ms, or briefly explain why the item is not applicable/relevant for your study

Sub-analyses by health literacy level is reported

18-i) Subgroup analysis of comparing only users

A subgroup analysis of comparing only users is not uncommon in ehealth trials, but if done, it must be stressed that this is a self-selected sample and no longer an unbiased sample from a randomized trial (see 16-iii).

	1	2	3	4	5	
subitem not at all important	○	○	◉	○	○	essential

Clear selection

Does your paper address subitem 18-i?

Copy and paste relevant sections from the manuscript (include quotes in quotation marks "like this" to indicate direct quotes from your manuscript), or elaborate on this item by providing additional information not in the ms, or briefly explain why the item is not applicable/relevant for your study

Not applicable - one off intervention

19) All important harms or unintended effects in each group
(for specific guidance see CONSORT for harms)

Does your paper address CONSORT subitem 19? *

Copy and paste relevant sections from the manuscript (include quotes in quotation marks "like this" to indicate direct quotes from your manuscript), or elaborate on this item by providing additional information not in the ms, or briefly explain why the item is not applicable/relevant for your study

Secondary outcomes included negative emotional response

19-i) Include privacy breaches, technical problems

Include privacy breaches, technical problems. This does not only include physical "harm" to participants, but also incidents such as perceived or real privacy breaches [1], technical problems, and other unexpected/unintended incidents. "Unintended effects" also includes unintended positive effects [2].

	1	2	3	4	5	
subitem not at all important	○	○	◉	○	○	essential

Clear selection

Does your paper address subitem 19-i?

Copy and paste relevant sections from the manuscript (include quotes in quotation marks "like this" to indicate direct quotes from your manuscript), or elaborate on this item by providing additional information not in the ms, or briefly explain why the item is not applicable/relevant for your study

Not applicable - no issues

19-ii) Include qualitative feedback from participants or observations from staff/researchers

Include qualitative feedback from participants or observations from staff/researchers, if available, on strengths and shortcomings of the application, especially if they point to unintended/unexpected effects or uses. This includes (if available) reasons for why people did or did not use the application as intended by the developers.

	1	2	3	4	5	
subitem not at all important	○	○	◉	○	○	essential

Clear selection

Does your paper address subitem 19-ii?

Copy and paste relevant sections from the manuscript (include quotes in quotation marks "like this" to indicate direct quotes from your manuscript), or elaborate on this item by providing additional information not in the ms, or briefly explain why the item is not applicable/relevant for your study

Limitations identified by researchers are discussed and qualitative responses from participants included; no space for additional reflections as 4 phases covered

DISCUSSION

22) Interpretation consistent with results, balancing benefits and harms, and considering other relevant evidence

NPT: In addition, take into account the choice of the comparator, lack of or partial blinding, and unequal expertise of care providers or centers in each group

22-i) Restate study questions and summarize the answers suggested by the data, starting with primary outcomes and process outcomes (use)

Restate study questions and summarize the answers suggested by the data, starting with primary outcomes and process outcomes (use).

	1	2	3	4	5	
subitem not at all important	○	○	○	○	○	essential

Does your paper address subitem 22-i? *

Copy and paste relevant sections from the manuscript (include quotes in quotation marks "like this" to indicate direct quotes from your manuscript), or elaborate on this item by providing additional information not in the ms, or briefly explain why the item is not applicable/relevant for your study

"Principal results
These results provide new insights into identifying and addressing behavioural barriers to COVID-19 testing, which is central to understanding and controlling COVID-19 and future pandemics. Phase 1 identified a wide range of barriers to COVID-19 testing that had not previously been described in the COVID-19 literature. These covered all three behavioural drivers in the COM-B model. Phase 2 found that the motivation and capability barriers were far more prevalent than opportunity barriers in Australia at the time of the study. Many barriers were reported as more prevalent amongst people with lower health literacy. Phases 3 and 4 tested different ways to address capability and motivation barriers. Phase 3 found no differences between standard government text about COVID-19 testing and a tailored text intervention that addressed many different barriers using health literacy design principles. Phase 4 found that audio-visual interventions to address key knowledge barriers are more effective than written government information for improving knowledge, but this was not enough to shift COVID-19 testing intentions in adjusted analyses. "

22-ii) Highlight unanswered new questions, suggest future research

Highlight unanswered new questions, suggest future research.

	1	2	3	4	5	
subitem not at all important	○	○	○	◉	○	essential

Clear selection

Does your paper address subitem 22-ii?

Copy and paste relevant sections from the manuscript (include quotes in quotation marks "like this" to indicate direct quotes from your manuscript), or elaborate on this item by providing additional information not in the ms, or briefly explain why the item is not applicable/relevant for your study

"Future research
The capability and motivation issues identified in this study apply to PCR testing, but there are likely to be additional barriers for rapid antigen testing (RAT) which were not approved in Australia at the time of the study. Different barriers encountered for RATs are being investigated in subsequent research, including individuals' ability to understand instructions, perform self-testing, and interpret the results correctly (e.g. see trial registration ACTRN12622001517763). Concerns have been raised about misinterpretation of negative results from RATs, which have a high error rate if the test is not used within the recommended time period after exposure to a COVID-19 case[50]. Another avenue for further work is to partner with the media to avoid the identification and stigmatisation of individuals with positive test results in future disease outbreaks[51]. Media reports and anecdotal data from frontline health professionals may be a useful way to quickly identify emerging local issues that could inform the measurement of testing barriers to make it more relevant to local communities."

20) Trial limitations, addressing sources of potential bias, imprecision, and, if relevant, multiplicity of analyses

20-i) Typical limitations in ehealth trials

Typical limitations in ehealth trials: Participants in ehealth trials are rarely blinded. Ehealth trials often look at a multiplicity of outcomes, increasing risk for a Type I error. Discuss biases due to non-use of the intervention/usability issues, biases through informed consent procedures, unexpected events.

	1	2	3	4	5	
subitem not at all important	○	○	○	○	◉	essential

Clear selection

Does your paper address subitem 20-i? *

Copy and paste relevant sections from the manuscript (include quotes in quotation marks "like this" to indicate direct quotes from your manuscript), or elaborate on this item by providing additional information not in the ms, or briefly explain why the item is not applicable/relevant for your study

"Strengths and limitations
This research began as an unfunded and rapidly developed response to the COVID-19 pandemic. By addressing methodological issues and building on the findings in each Phase, we were able to better target the final intervention and show the value of audio-visual formats to address common knowledge barriers among people with varying health literacy needs.
Phase 1 identified the range of barriers to COVID-19 testing in Australia for the first time, but prevalence of the most important barriers could not be ascertained from these findings due to the reliance on open responses and a non-representative sample. The next phase aimed to address these methodological issues. Phase 2 identified the prevalence of COVID-19 testing barriers in a nationally representative sample, and highlighted important health literacy disparities. However, even the second phase was not representative of all community groups, particularly those from culturally and linguistically diverse backgrounds which has been identified as a key area of need in Australia and worldwide. We have conducted a separate survey with these groups using interpreters to conduct the survey in Phase 1 in preferred languages, as a partnership with Western Sydney Local health District[52]–[54].

In Phase 3, the intervention's highly tailored design meant that there was considerable heterogeneity in the intervention elements that participants received. This may have contributed to the lack of observed effect. It is possible there were ceiling effects for testing intentions when participants assumed they would get tested when they were not currently or recently thinking about the logistics of getting tested. It is also possible that participants did not engage with the text-based intervention content. We attempted to address these methodological issues in the final Phase 4 by focusing on a consistent set of key knowledge barriers in a more targeted group with lower baseline testing intentions (younger, lower education), using a more engaging intervention format (animation with text and audio, and a social media style video), and including more sensitive measures of testing intention to avoid potential biases.

The audio-visual interventions produced for Phase 4 have information that is specific to the Australian context so may not be useful in other countries, but can be used as a starting point for new knowledge interventions. We found that a simple and relatively cheap animation focused on key messages or a Tiktok style video that incorporated humour were both effective for increasing knowledge but not testing intentions. However, the findings may not be generalisable to other contexts, particularly where opportunity issues such as cost or physical access to testing is a problem. We expect there will be additional barriers to rapid antigen tests. Nevertheless, this paper provides a comprehensive list of testing barriers that may help us better prepare for future variants or the next pandemic. "

21) Generalisability (external validity, applicability) of the trial findings
NPT: External validity of the trial findings according to the intervention, comparators, patients, and care providers or centers involved in the trial

21-i) Generalizability to other populations

Generalizability to other populations: In particular, discuss generalizability to a general Internet population, outside of a RCT setting, and general patient population, including applicability of the study results for other organizations

	1	2	3	4	5	
subitem not at all important	○	○	○	◉	○	essential

Clear selection

Does your paper address subitem 21-i?

Copy and paste relevant sections from the manuscript (include quotes in quotation marks "like this" to indicate direct quotes from your manuscript), or elaborate on this item by providing additional information not in the ms, or briefly explain why the item is not applicable/relevant for your study

"The audio-visual interventions produced for Phase 4 have information that is specific to the Australian context so may not be useful in other countries, but can be used as a starting point for new knowledge interventions. We found that a simple and relatively cheap animation focused on key messages or a Tiktok style video that incorporated humour were both effective for increasing knowledge but not testing intentions. However, the findings may not be generalisable to other contexts, particularly where opportunity issues such as cost or physical access to testing is a problem. We expect there will be additional barriers to rapid antigen tests. Nevertheless, this paper provides a comprehensive list of testing barriers that may help us better prepare for future variants or the next pandemic. "

21-ii) Discuss if there were elements in the RCT that would be different in a routine application setting

Discuss if there were elements in the RCT that would be different in a routine application setting (e.g., prompts/reminders, more human involvement, training sessions or other co-interventions) and what impact the omission of these elements could have on use, adoption, or outcomes if the intervention is applied outside of a RCT setting.

	1	2	3	4	5	
subitem not at all important	○	○	◉	○	○	essential

Clear selection

Does your paper address subitem 21-ii?

Copy and paste relevant sections from the manuscript (include quotes in quotation marks "like this" to indicate direct quotes from your manuscript), or elaborate on this item by providing additional information not in the ms, or briefly explain why the item is not applicable/relevant for your study

Not applicable - one off intervention

OTHER INFORMATION

23) Registration number and name of trial registry

Does your paper address CONSORT subitem 23? *

Copy and paste relevant sections from the manuscript (include quotes in quotation marks "like this" to indicate direct quotes from your manuscript), or elaborate on this item by providing additional information not in the ms, or briefly explain why the item is not applicable/relevant for your study

"the experiments were pre-registered on the Australia New Zealand Trial Registry (ACTRN12621000876897[16]; ACTRN12620001355965[17]). "

24) Where the full trial protocol can be accessed, if available

Does your paper address CONSORT subitem 24? *

Cite a Multimedia Appendix, other reference, or copy and paste relevant sections from the manuscript (include quotes in quotation marks "like this" to indicate direct quotes from your manuscript), or elaborate on this item by providing additional information not in the ms, or briefly explain why the item is not applicable/relevant for your study

"the experiments were pre-registered on the Australia New Zealand Trial Registry (ACTRN12621000876897[16]; ACTRN12620001355965[17])."

25) Sources of funding and other support (such as supply of drugs), role of funders

Does your paper address CONSORT subitem 25? *

Copy and paste relevant sections from the manuscript (include quotes in quotation marks "like this" to indicate direct quotes from your manuscript), or elaborate on this item by providing additional information not in the ms, or briefly explain why the item is not applicable/relevant for your study

"The surveys were not specifically funded, but in-kind support was provided by authors with research fellowships. The experimental trials were funded by the Marie Bashir Institute (now named the Sydney Institute for Infectious Diseases) at the University of Sydney.
Carissa Bonner is supported by a National Health and Medical Research Council (NHMRC)/Heart Foundation Early Career Fellowship (#1122788).
Kirsten McCaffery is supported by a National Health and Medical Research Council (NHMRC) Principal Research Fellowship (#1121110)."

X27) Conflicts of Interest (not a CONSORT item)

X27-i) State the relation of the study team towards the system being evaluated

In addition to the usual declaration of interests (financial or otherwise), also state the relation of the study team towards the system being evaluated, i.e., state if the authors/evaluators are distinct from or identical with the developers/sponsors of the intervention.

	1	2	3	4	5	
subitem not at all important	○	○	◉	○	○	essential

Clear selection

Does your paper address subitem X27-i?

Copy and paste relevant sections from the manuscript (include quotes in quotation marks "like this" to indicate direct quotes from your manuscript), or elaborate on this item by providing additional information not in the ms, or briefly explain why the item is not applicable/relevant for your study

"We thank the participants of the longitudinal COVID-19 survey for their ongoing participation in this research, Wendy Liang for consumer input for the written intervention, and Mustafa Dhahir for developing the Tiktok version of the intervention."

About the CONSORT EHEALTH checklist

As a result of using this checklist, did you make changes in your manuscript? *

- ○ yes, major changes
- ○ yes, minor changes
- ◉ no

What were the most important changes you made as a result of using this checklist?

Your answer

How much time did you spend on going through the checklist INCLUDING making changes in your manuscript *

one hour after acceptance - not really applicable to the multi phase paper

As a result of using this checklist, do you think your manuscript has improved? *

- ○ yes
- ◉ no
- ○ Other:

Would you like to become involved in the CONSORT EHEALTH group?

This would involve for example becoming involved in participating in a workshop and writing an "Explanation and Elaboration" document

- ○ yes
- ◉ no
- ○ Other:

Clear selection

Any other comments or questions on CONSORT EHEALTH

Your answer

STOP - Save this form as PDF before you click submit

To generate a record that you filled in this form, we recommend to generate a PDF of this page (on a Mac, simply select "print" and then select "print as PDF") before you submit it.

When you submit your (revised) paper to JMIR, please upload the PDF as supplementary file.

Don't worry if some text in the textboxes is cut off, as we still have the complete information in our database. Thank you!

Final step: Click submit !
Click submit so we have your answers in our database!

Submit

Clear form

Never submit passwords through Google Forms.

This content is neither created nor endorsed by Google. Report Abuse - Terms of Service - Privacy Policy

Google Forms
